# Photoelectrochemical Coupling of Waste‐Nitrogen Oxidation Reactions with Hydrogen Evolution for Sustainable Energy Conversion

**DOI:** 10.1002/cssc.202501772

**Published:** 2025-11-24

**Authors:** Maheswari Arunachalam, Jyoti Ganapati Badiger, Suzan Abdelfattah Sayed, Soon Hyung Kang

**Affiliations:** ^1^ Department of Chemistry Education Chonnam National University Gwangju Republic of Korea; ^2^ Department of Interdisciplinary Program for Photonic Engineering Chonnam National University Gwangju Republic of Korea; ^3^ Department of Advanced Chemicals and Engineering Chonnam National University Gwangju Republic of Korea

**Keywords:** ammonia oxidation, NO_x_ oxidation, photoelectrochemical water splitting, solar fuel conversion, urea oxidation

## Abstract

Photoelectrochemical (PEC) water splitting offers a sustainable method for hydrogen production, but is limited by slow oxygen evolution reaction (OER) kinetics and the low economic value of oxygen (O_2_). Alternative anodic oxidation reactions have been developed to replace OER, enhancing energy efficiency and producing valuable products. This review analyzes recent advancements in photoanodes for the selective oxidation of urea, ammonia, and nitrogen oxides under solarlight into valuable chemicals,such as nitrogen (N_2_), carbon dioxide (CO_2_), and nirtates, by utilizing alternative oxidation pathways alongside the hydrogen evolution reaction (HER). This review focuses on the mechanistic pathways of oxidation, highlighting strategies to tackle challenges such as incomplete oxidation and nitrate buildup through optimized catalyst design, nanostructuring, and interfacial engineering. Key systems include nickel phosphide (Ni_2_P)‐sensitized titanium dioxide (TiO_2_) nanotubes, silicon (Si) photoanodes with Ni‐based cocatalysts, and amorphous Ni–Mo–O layers, all showing better charge separation, lower overpotentials, and strong long‐term stability. Additionally, PEC NO oxidation provides a low‐temperature, selective approach for transforming trace NO pollutants into nitrates suitable for fertilizer, supported by reactor‐scale innovations in gas‐phase PEC systems. This review examines catalyst stability, selectivity, and device design, suggesting future directionsfor scalable, durable, and affordable PEC systems that promote clean energy and environmental sustainability.

## Graphical abstract

Photoelectrochemical (PEC) cells utilize alternative oxidation pathways, allowing for the production of high‐value products, including N_2_, CO_2_, and nitrates. Key advantages include innovative catalyst design, improved charge separation, reduced energy input, and economic viability. Through nanostructured and engineered photoanodes, PEC cells achieve multifunctional performance—simultaneously enabling clean energy production.

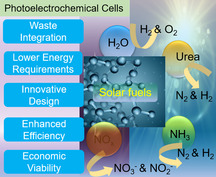

## 1 Introduction

Over the last hundred years, fossil fuels like coal, oil, and natural gas have been the primary energy sources because of their affordability and widespread availability [1, 2, 3, 4]. However, they are the main contributors to human‐made carbon dioxide (CO_2_) emissions and are running out. This leads to increasing environmental issues such as global warming, pollution, energy insecurity, and harm to our ecosystems [5]. Shifting to more sustainable and environmentally friendly resources is a strategic goal for the energy industry. Consequently, hydrogen (H_2_) has gained significant global attention as a clean, promising energy carrier to decrease reliance on fossil fuels gradually. Being the most abundant element on Earth, hydrogen boasts a high gravimetric energy density of 120 MJ kg^−1^ at a lower heating value, which is much higher than petrol (2 MJ kg^−1^) and diesel (45.3 MJ kg^−1^) [6, 7]. Additionally, hydrogen can be liquefied or transformed into other carriers, allowing it to be used within existing infrastructure across various sectors like transportation, agriculture, heat, and electricity. Consequently, hydrogen is expected to become a significant energy carrier for a sustainable energy economy [8, 9, 10, 11, 12, 13, 14]. Meanwhile, solar energy supplies around 1.7 × 10^17^ watts to Earth's surface, making it the most abundant and accessible renewable resource. Advances in photovoltaic technology—alongside progress in wind, hydro, and geothermal energy—are already helping renewables replace some fossil fuel‐based power and cut greenhouse gas emissions. However, the fluctuating supply of sunlight and the high cost of storing large amounts of electricity remain challenges. Therefore, current research aims to investigate technologies capable of directly converting solar photons into stable and easily transportable energy carriers, with hydrogen as a primary target.

### 1.1 Solar‐Powered Electrochemical Reaction

Solar energy drives various electrochemical processes that generate multiple energy fuels through simple reduction and oxidation reactions [15]. Major products include hydrogen gas (H_2_), formic acid (HCOOH), carbon monoxide (CO), hydrocarbons (C_2_), and ammonia (NH_3_), as well as nitrogen oxides like O_2_, N_2_, NO_2_^−^, and NO_3_^−^. These substances serve as clean energy carriers, industrial feedstocks, and chemical precursors, emphasizing the versatility and potential of solar‐driven electrochemical systems in advancing sustainable energy and chemical production. This approach can decrease reliance on fossil fuels and promote a cleaner energy future [16, 17, 18, 19]. Currently, development of solar–powered techniques is actively progressing, which can be broadly classified into three main categories.

• Photovoltaic‐electrolysis (PV–EC) combines solar panels with standalone electrolyzers and is commercially mature. Voltage‐matching losses limit its overall efficiency, as well as the need for additional balance‐of‐system hardware and separate gas‐storage units.
• Photocatalysis involves catalyst powders dispersed in a reactor, making the concept straightforward. However, practical devices currently achieve sub‐1% quantum efficiencies, which hampers large‐scale use.
• PEC cells uniquely combine light absorption and catalysis within a single semiconductor–electrolyte junction. Sunlight creates electron–hole pairs inside the immersed semiconductor, and these charge carriers then facilitate redox reactions at the interface, converting abundant feedstock like water or small organics into hydrogen and other valuable chemicals under ambient conditions [20, 21, 22, 23, 24, 25, 26, 27, 28, 29, 30].

Although PV‐EC, photocatalysis, and PEC cells each have clear benefits, the four‐electron oxygen‐evolution reaction (OER) at the photoanode remains the primary challenge due to its kinetic and energy hurdles in solar‐to‐hydrogen conversion. OER demands a significant overpotential‐often hundreds of millivolts above the 1.23 V thermodynamic threshold‐and involves multiple proton‐coupled electron transfers. These factors collectively suppress photocurrent, accelerate photo‐corrosion, and shorten electrode lifespan. As a result, recent PEC research is increasingly exploring alternative anodic reactions, such as the selective oxidation of urea, ammonia, or NO_*X*_, which happen at much lower potentials and with faster reaction rates. Table 1 summarizes key target reactions in PEC systems and their conversion into valuable products.

**TABLE 1:** Summary of nitrogen based oxidation reactions in PEC systems and the corresponding value products obtained through these processes.

Oxidation Reaction	Potential (*V*_RHE_)	Key Points	Final Products
Urea oxidation	≈0.37 V	≈0.9 V lower than OER Rapid kinetics Environmentally friendly	N_2_ gas Carbon dioxide (CO_2_)
Ammonia oxidation	≈0.06 V (to N_2_)	Six‐electron process Very low overpotential bias High efficiency	N_2_ gas Additional H_2_ gas as a co‐product
NO/NO_2_ oxidation	Near 0 V (NO → NO_3_^−^)	Applicable in gas and liquid phases Selective oxidation Effective for emission control	Nitrate fertilizer (NO _3_^−^)

### 1.2 Alternative PEC Oxidation

Alternative anodic reactions in PEC cells, such as the oxidation of urea, ammonia, and nitrogen oxides, occur at much lower overpotentials than the OER. They also transform pollutants into valuable products. The semiconductor's ability to generate an internal photovoltage and to separate redox sites cleanly allows PEC cells to avoid the high external bias needed in purely electrochemical systems and reduces charge‐recombination losses common in particle photocatalysis. Using these nitrogen‐based pathways instead of OER increases photocurrent density, enhances solar‐to‐fuel efficiency, and enables the combination of hydrogen (or other fuel) production with wastewater and flue‐gas cleanup—providing a scalable, cost‐effective approach to sustainable fuels and chemicals [31, 32, 33].

Commercialization and industrialization of PEC systems are vital to translate laboratory advances into practical applications. Beyond academic interest, PEC systems provide scalable and sustainable hydrogen production while simultaneously removing nitrogen‐based pollutants such as urea, ammonia, and NO_*X*_. This dual benefit reduces energy use compared to conventional OER, lowers costs, and generates valuable byproducts. For industries including wastewater treatment, fertilizer recovery, and clean fuel production, achieving high PEC efficiency enhances competitiveness and supports circular economy goals.

For efficiency, targets must be carefully addressed in terms of both energy conversion and overall system viability. Traditional PEC water splitting is limited by the sluggish OER, which demands high overpotentials. By substituting OER with UOR, AOR, or NO oxidation, the thermodynamic voltage requirements can be reduced significantly (0.37 V for UOR and 0.06 V for AOR, compared to 1.23 V for OER), directly improving solar to hydrogen (STH) efficiency. For real‐world deployment, however, laboratory‐scale demonstrations of 1%–3% STH efficiency are insufficient. A practical PEC system must achieve an STH efficiency of greater than 10%, aligning with Department of Energy benchmarks for solar fuels, while maintaining stability over more than 1000 h of continuous operation. PEC UOR is probably the most practical option for now. Industrial and municipal wastewater contain high amounts of urea, making it a cheap feedstock while also helping with environmental cleanup. UOR also lowers the required anodic potential, and Ni‐ and Co‐based catalysts have already reached photocurrent densities close to what is needed for about 8% STH efficiency. With further progress, going beyond 10% seems possible soon [34].

PEC AOR has the advantage to need the lower theoretical voltage, which enhances its efficiency. Nonetheless, ammonia‐rich wastewater is less common than urea‐containing effluents, and the oxidation process faces challenges from competing pathways that produce unwanted byproducts like N_2_O. Although advanced catalysts can boost selectivity, achieving stable systems with over 10% STH efficiency through AOR is probably a mid‐term objective rather than an immediate goal. NO oxidation remains the most challenging route. Despite its potential for reducing NO_*X*_ emissions, the reaction mechanism is complex, involving multiple reactive oxygen species and intermediates. Current PEC efficiencies are still low, and durability in realistic flue gas conditions is not established. To achieve >10% efficiency with >90% selectivity toward nitrate/nitrite products, significant advances in catalyst design and reactor engineering will be required [35, 36].

**SCHEME 1:**
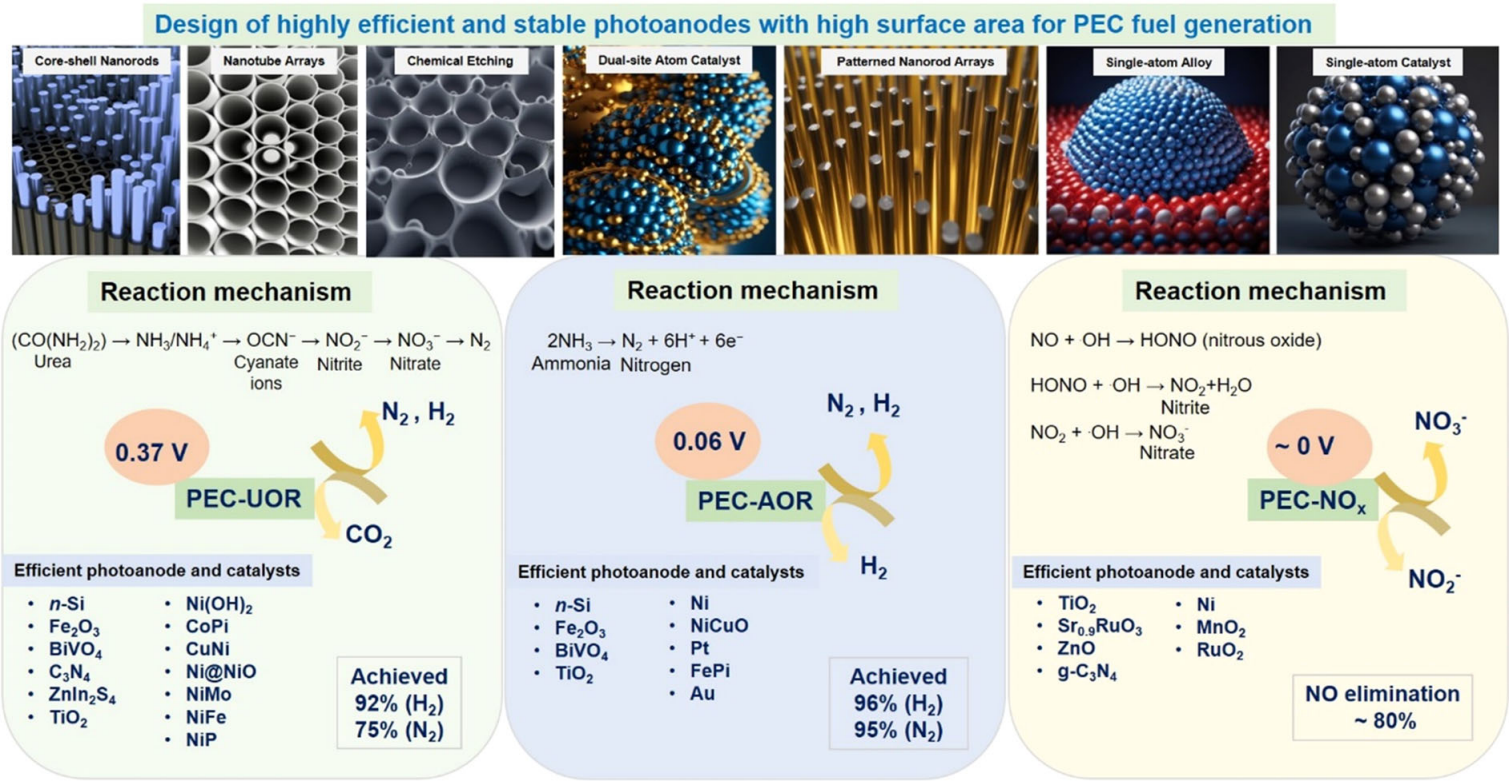
Schematic diagram showing representative photoanode designs and their associated reaction mechanisms for urea oxidation, ammonia oxidation, and NO_*X*_ oxidation, along with their connection to hydrogen evolution.

This review summarizes recent progress in developing advanced photoanodes for the oxidation of urea, ammonia, and NO_*x*_ in PEC systems. It explains how these alternative anodic reactions reduce overpotentials, improve reaction kinetics, and transform pollutants into valuable chemicals, effectively integrating waste treatment with hydrogen production. The review explores mechanistic insights, material design strategies, and performance enhancements to guide future research. Overall, waste‐nitrogen oxidation can increase solar‐to‐fuel efficiency and provide environmental benefits. The following sections cover each waste‐nitrogen pathway—Section 2 on urea, Section 3 on ammonia, and Section 4 on nitrogen oxides—highlighting recent PEC‐based oxidation methods, from mechanistic understanding and catalyst development to device performance improvements (Scheme 1).

## 2 PEC Urea Oxidation (PEC UOR)

PEC UOR usually involves a semiconductor photoanode exposed to light, which causes urea to oxidize into products like N_2_, CO_2_, and water. This process includes several intermediate products. Initially, the photogenerated holes significantly speed up urea breakdown, leading to quicker and more efficient formation of intermediates. Specifically, urea decomposes into ammonia (NH_3_) and cyanate ions (OCN^−^), triggering a series of oxidation steps outlined in (Equation 1). Typically, this process involves the simultaneous oxidation of nitrogen—through intermediates such as NH_3_, OCN^−^, NO_2_^−^, and NO_3_^−^—and carbon—via cyanate (OCN^−^), carbonate (CO_3_^2−^), and bicarbonate (HCO_3_^−^)—due to the presence of both C and N atoms in urea (see (Equations 2) and (3)).

(1) $$\mathrm{CO}{\left({\mathrm{NH}}_{2}\right)}_{2}+2h^{+}+2{\mathrm{OH}}^{-}\to {{\mathrm{NH}}_{3}}^{+}{\mathrm{OCN}}^{-}+H_{2}O$$

Typical overall reaction pathway (simplified):

(2) $$(\mathrm{CO}{\left({\mathrm{NH}}_{2}\right)}_{2})\to {\mathrm{NH}}_{3}/{{\mathrm{NH}}_{4}}^{+}\to {\mathrm{OCN}}^{-}\to {{\mathrm{NO}}_{2}}^{-}\to {{\mathrm{NO}}_{3}}^{-}\to N_{2}$$

or

(3) $$(\mathrm{CO}{\left({\mathrm{NH}}_{2}\right)}_{2})\to {\mathrm{OCN}}^{-}\to {{\mathrm{CO}}_{3}}^{2-}/{{\mathrm{HCO}}_{3}}^{-}\to {\mathrm{CO}}_{2}$$

In PEC UOR, NO_2_^−^ ions are highly reactive intermediates, quickly oxidized by photogenerated holes (h^+^) and reactive radicals such as ·OH, resulting in stable compounds like nitrate (NO_3_^−^) or harmless N_2_ under PEC oxidative conditions (see (Equation 6) and (7)). Usually, NO_2_^−^ forms as an intermediate during the partial oxidation of ammonia (NH_3_/NH_4_^+^) or other nitrogenous intermediates like OCN^−^ (see (Equation 4) and (5)). In here, the NH_3_ first oxidizes to NO_2_^−^ ions, due to incomplete oxidation, and typically represents a transient state before further oxidation.

(4) $${\mathrm{NH}}_{3}+3{\mathrm{OH}}^{-}+2h^{+}\to {{\mathrm{NO}}_{2}}^{-}+3H_{2}O$$

(5) $${\mathrm{OCN}}^{-}+4{\mathrm{OH}}^{-}+4h^{+}\to {{\mathrm{NO}}_{2}}^{-}+{\mathrm{CO}}_{2}+2H_{2}O$$

(6) $${{\mathrm{NO}}_{2}}^{-}+2{\mathrm{OH}}^{-}+h^{+}\to {{\mathrm{NO}}_{3}}^{-}+H_{2}O$$

(7) $$2{{\mathrm{NO}}_{2}}^{-}+4H^{+}\to N_{2}+2O_{2}$$

According to the typical reaction pathways, NH_3_, initially produced by partial hydrolysis (Equation 1) or oxidation of urea, undergoes further oxidation to nitrite ions (NO_2_^−^) (Equation 4). Then, nitrite is oxidized to nitrate (NO_3_^−^). While nitrate is stable, its buildup is problematic because nitrate is an environmental pollutant that can cause eutrophication in aquatic ecosystems by encouraging excessive algal growth and oxygen depletion. Nitrate buildup indicates incomplete oxidation, caused by insufficient generation or slow transfer of photogenerated holes or hydroxyl radicals (·OH). This results in premature stopping of the reaction at nitrate, rather than complete conversion to environmentally safe N_2_. High nitrate levels highlight the need to improve PEC system design, either by enhancing catalyst design or by optimizing the overall PEC process [35, 36, 37].

Under mild oxidative conditions, NH_3_ or nitrogenous intermediates can undergo partial oxidation or coupling reactions to form hydrazine (N_2_H_4_). This is particularly possible if reaction conditions and catalysts favor N—N bond formation rather than complete oxidation to NO_2_^−^ or NO_3_^−^. For example, hydrazine can be formed via the coupling of radical intermediates derived from ammonia (Equation 8):

(8) $$2{\mathrm{NH}}_{3}+2h^{+}\to N_{2}H_{4}+2H^{+}$$

Such reactions typically occur at lower oxidation potentials or in catalytic environments that favor N—N coupling instead of complete oxidative cleavage.

N_2_H_4_ is highly reactive, rapidly oxidizing further under PEC conditions to N_2_, NO_2_^−^, or NO_3_^−^ (Equation 9) and (10):

(9) $$N_{2}H_{4}+4{\mathrm{OH}}^{-}+4h^{+}\to N_{2}+4H_{2}O$$

(10) $$N_{2}H_{4}+8{\mathrm{OH}}^{-}+8h^{+}\to 2{{\mathrm{NO}}_{3}}^{-}+6H_{2}O$$

Lower oxidation potentials or catalytic environments promoting partial oxidation or N—N coupling significantly increase N_2_H_4_ formation.

Meanwhile, the carbonate (CO_3_^2−^) and bicarbonate (HCO_3_^−^) ions typically form as intermediates during the oxidation of urea. During PEC oxidation, the carbon backbone of urea undergoes stepwise oxidation (Equation 11) and (12):

(11) $$\mathrm{Urea}\to \mathrm{Cyanate}({\mathrm{OCN}}^{-})\to \mathrm{Carbonate}({{\mathrm{CO}}_{3}}^{2-})/\mathrm{Bicarbonate}({{\mathrm{HCO}}_{3}}^{-})\to {\mathrm{CO}}_{2}$$

(12) $${\mathrm{OCN}}^{-}+3{\mathrm{OH}}^{-}+2h^{+}\to {{\mathrm{CO}}_{3}}^{2-}+{\mathrm{NH}}_{3}+H_{2}O$$

CO_3_^2−^ and HCO_3_^−^ ions are stable intermediates that form when organic carbon is not fully oxidized into CO_2_. In alkaline conditions, CO_3_^2−^ ions are thermodynamically favored, whereas HCO_3_^−^ ions predominate in neutral or mildly acidic conditions. These CO_3_^2−^ and HCO_3_^−^ ions can undergo further oxidation under PEC conditions, ultimately yielding CO_2_ as the final stable oxidation product (Equation 13) and (14).

(13) $${{\mathrm{CO}}_{3}}^{2-}+2h^{+}\to {\mathrm{CO}}_{2}+1/2O_{2}$$

or under acidic conditions (protonation and decomposition):

(14) $${{\mathrm{HCO}}_{3}}^{-}+h^{+}\to {\mathrm{CO}}_{2}+1/2H_{2}O+1/4O_{2}$$

In summary, both nitrogen and carbon oxidation pathways occur together during PEC urea oxidation. This process effectively transforms nitrogen into N_2_ and carbon into CO_2_, fully oxidizing urea into harmless products. The presence of these dual pathways highlights the importance of optimizing reaction conditions and catalyst design to achieve complete oxidation of nitrogen‐ and carbon‐containing intermediates, preventing the formation of partial byproducts like nitrate or carbonate.

### 2.1 PEC Urea Oxidation Over Conventional Water Electrolysis

Urea is a hydrogen‐rich, water‐soluble compound that is widely available and derived from sources like fertilizers, urine, and industrial waste. Compared to traditional water oxidation, PEC urea oxidation provides several significant benefits. Recent advances in electrochemical UOR further illustrate how catalyst design governs both activity and selectivity. Studies on Ni‐based systems—such as phosphorus substitution in NiCo_2_O_4_, lattice oxygen activation in LiNiO_2_, and sulfur incorporation in NiMoO_4_—demonstrate that electronic delocalization, lattice oxygen participation, and dopant incorporation are powerful strategies to modulate reactivity and product distribution. These principles are directly relevant to NO_*X*_ oxidation, where similar design strategies can guide the development of efficient and selective photoanodes [15, 38, 39]. Notably, the thermodynamic onset potential of UOR (≈0.37 V vs. RHE) is much lower than that of OER, which generally requires 1.6–2.0 V in practical PEC setups. These advantages emerge by adding urea to the electrolyte, allowing for efficient PEC oxidation at lower overpotentials. This leads to reduced cell voltages, improved energy efficiency, and increased hydrogen output. Beyond energy benefits, PEC‐UOR offers environmental advantages by removing urea from wastewater and converting it into harmless products like nitrogen and carbon dioxide instead of O_2_, making the process safer and more energy‐efficient. This integrated approach—combining pollution removal with solar hydrogen generation—is challenging to achieve with traditional electrochemical or photocatalytic systems alone. Recent studies employing advanced photoanodes such as Ni‐doped TiO_2_, BiVO_4_, and metal–insulator–semiconductor (MIS)‐structured Si electrodes have demonstrated significant improvements in activity, selectivity, and stability, making PEC‐UOR a feasible and scalable solution for sustainable energy and environmental applications. However, effective PEC UOR also depends on careful photoelectrode design. Although low‐bandgap semiconductors enhance sunlight absorption, they often suffer from poor carrier mobility and short diffusion lengths, resulting in higher recombination losses. To address these issues, approaches such as bandgap engineering, heterojunction creation, and nanostructuring are extensively studied. Nanoscale architectures, in particular, improve light capture, increase the electrochemically active surface area, and facilitate charge separation—ultimately enhancing the overall PEC performance for UOR.

### 2.2 Nanostructuring of Semiconductor Surfaces: Ni_2_P‐Sensitized TiO_2_ Nanotube Arrays

**FIGURE 1:**
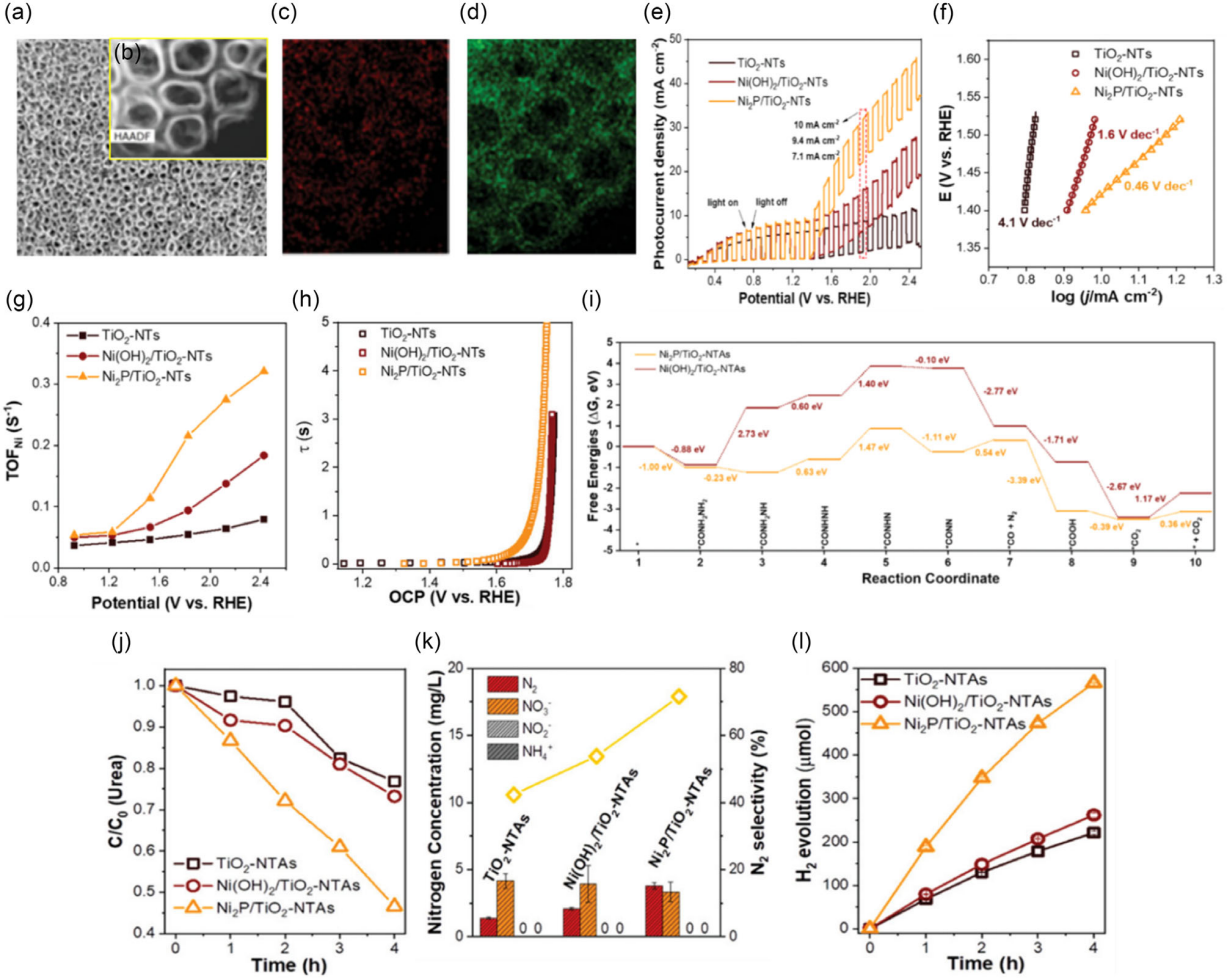
(a) FE‐SEM image, (b) TEM image of Ni_2_P/TiO_2_‐NTAs, (c,d) mapping of HAADF–STEM images of Ni_2_P in TiO_2_‐NTAs, (e) photocurrent‐potential curves under chopped illumination, (f) Tafel slopes, (g) TOF values during the UOR process, (h) electron lifetimes, (i) Gibbs free energy (Δ*G*) diagrams of the various intermediate species along the reaction pathways of UOR on Ni_2_P/TiO_2_‐NTAs and Ni(OH)_2_/TiO_2_‐NTAs surfaces, (j) urea removal, (k) concentration of produced nitrogen species, and (l) H_2_ evolution rate on different photoanodes. Reproduced with permission [41]. Copyright 2023, Wiley‐VCH.

Urea‐rich industrial wastewater presents a dual opportunity: it poses a significant environmental challenge while also offering a potential energy source for sustainable hydrogen production. This study presents a semiconductor/cocatalyst photoanode—Ni_2_P cluster‐sensitized TiO_2_ nanotube arrays (Ni_2_P/TiO_2_‐NTAs)—designed to efficiently drive the six‐electron PEC‐UOR, combining water purification with solar‐assisted hydrogen production [40, 41]. Vertically aligned TiO_2_ nanotubes (about 910 nm long, 28 nm diameter) were made through anodization and then seeded with Nanometer‐scale Ni(OH)_2_, which was phosphatized to produce uniform ≈3 nm Ni_2_P clusters ([Field‐Emission Scanning Electron Microscopy (FE‐SEM), High‐Resolution Transmission Electron Microscopy (HR‐TEM) and elemental maps shown in Figure 1a–d]. This structure creates close Ni_2_P/TiO_2_ junctions, a large surface area, and direct diffusion channels, all while maintaining the scaffold's optical transparency. Under 365 nm light in 1 M KOH + 0.5 M urea, the photoanode reaches 10 mA·cm^−2^ at only 1.43 V versus RHE—an 810 and 150 mV reduction compared to pristine TiO_2_‐NTAs and Ni(OH)_2_/TiO_2_‐NTAs, respectively (Figure 1e). The Tafel slope (0.46 V dec^−1^), carrier lifetime (8.9 ms), and turnover frequency (TOF) efficiency all outperform current standards, indicating faster charge transfer at the interface and reduced recombination (Figure 1f–h).

As demonstrated by density functional theory (Figure 1i), the enhanced performance results from the synergistic interaction between surface chemistry and electronic properties. Ni_2_P shows stronger urea adsorption (*E*_ads_ = −0.78 eV) and significantly lower CO_2_ desorption energy (0.19 eV) compared to Ni(OH)_2_, which promotes N—H dehydrogenation and prevents active site blockage. Experiments reveal that the electrode removes 53% of urea and 41% of total nitrogen in 4 h, achieving 71.6% of N_2_ selectivity, and maintaining 67% of its photocurrent after 10 h (Figure 1j‐k). It exhibits the fastest hydrogen evolution among the control samples and functions as a photo‐assisted anode in a direct urea fuel cell, delivering an open‐circuit voltage (*V*_OC_) of 0.63 V, a short‐circuit current density (*J*_SC_) of 82 µA cm^−2^, and a maximum power output (*P*_max_) of 28.5 µW cm^−2^, as shown in Figure 1l. Ni_2_P/TiO_2_‐NTAs thus serve as a prime example of how integrating nanoscale phosphide cocatalysts with structured oxide photoanodes can effectively reduce urea oxidation overpotentials, accelerate interfacial charge transfer, and enable the dual functions of wastewater treatment and sustainable hydrogen production. Consequently, the Ni_2_P/TiO_2_‐NTAs system illustrates how precise nanoscale design can simultaneously overcome kinetic limitations, improve reaction selectivity, and enable efficient pollutant removal under practical operating conditions. These findings highlight the potential of rationally engineered photoanodes that combine nanostructured semiconductors with molecularly tailored cocatalysts to achieve high‐efficiency PEC urea oxidation.

For instance, Ni_6_MnO_8_ nanostructures (5–10 nm) provide abundant Ni^2+^/Ni^3+^ redox sites, shortened charge‐transport pathways, and enhanced porosity. These features overall suppress charge recombination, improve adsorption/diffusion of urea molecules, and accelerate reaction kinetics, resulting in superior activity and durability compared with bulk counterparts. Similarly, the construction of a 2D/2D WO_3_/*g*‐C_3_N_4_ heterostructure integrated with porous Ni@CF demonstrates how interfacial engineering and hierarchical porosity can synergistically promote charge separation and mass transport. The intimate contact between WO_3_ and *g*‐C_3_N_4_ suppresses electron–hole recombination, while conductive Ni‐decorated carbon felt provides efficient electron pathways and large surface area for active sites. This strategy not only reduced the applied potential for urea splitting (from 1.80 to 1.18 V) but also enabled stable H_2_ production for 18 h, highlighting its potential for long‐term operation. Another notable example is theNi(OH)_2_‐modified Ti‐doped α‐Fe_2_O_3_ system, where the Ni(OH)_2_ overlayer serves as a hole‐storage layer that prolongs carrier lifetime, reduces recombination, and supplies abundant surface‐active sites. This dual functionality markedly improved PEC urea oxidation, lowering the onset potential by 100 mV and enhancing photocurrent density by fourfold compared with the unmodified photoanode.^[^ ^42^, ^43^, ^44^ ^]^

### 2.3 Mitigating Trap‐Assisted Recombination via Carrier Selectivity at MIS Interfaces

Silicon (Si) is a prominent material for PEC device photoanodes because of its high absorption of the solar spectrum, low cost, widespread manufacturing methods, and naturally long carrier diffusion lengths. However, unmodified Si tends to corrode easily in aqueous electrolytes and shows only moderate catalytic activity for the UOR. To overcome these issues, scientists have used ultrathin passivation layers and integrated efficient catalysts. In particular, metal–insulator–semiconductor (MIS) architectures have gained attention. In this setup, a conformal insulator—usually native silicon oxide or a deposited dielectric—serves two functions: it chemically shields the Si surface from corrosion. It spatially isolates the semiconductor from the metal catalyst, preventing metal‐induced gap states that cause nonradiative recombination. Consequently, MIS‐structured Si photoanodes demonstrate improved charge separation, less surface recombination, and higher photovoltage, leading to better PEC UOR performance under sunlight.

**FIGURE 2:**
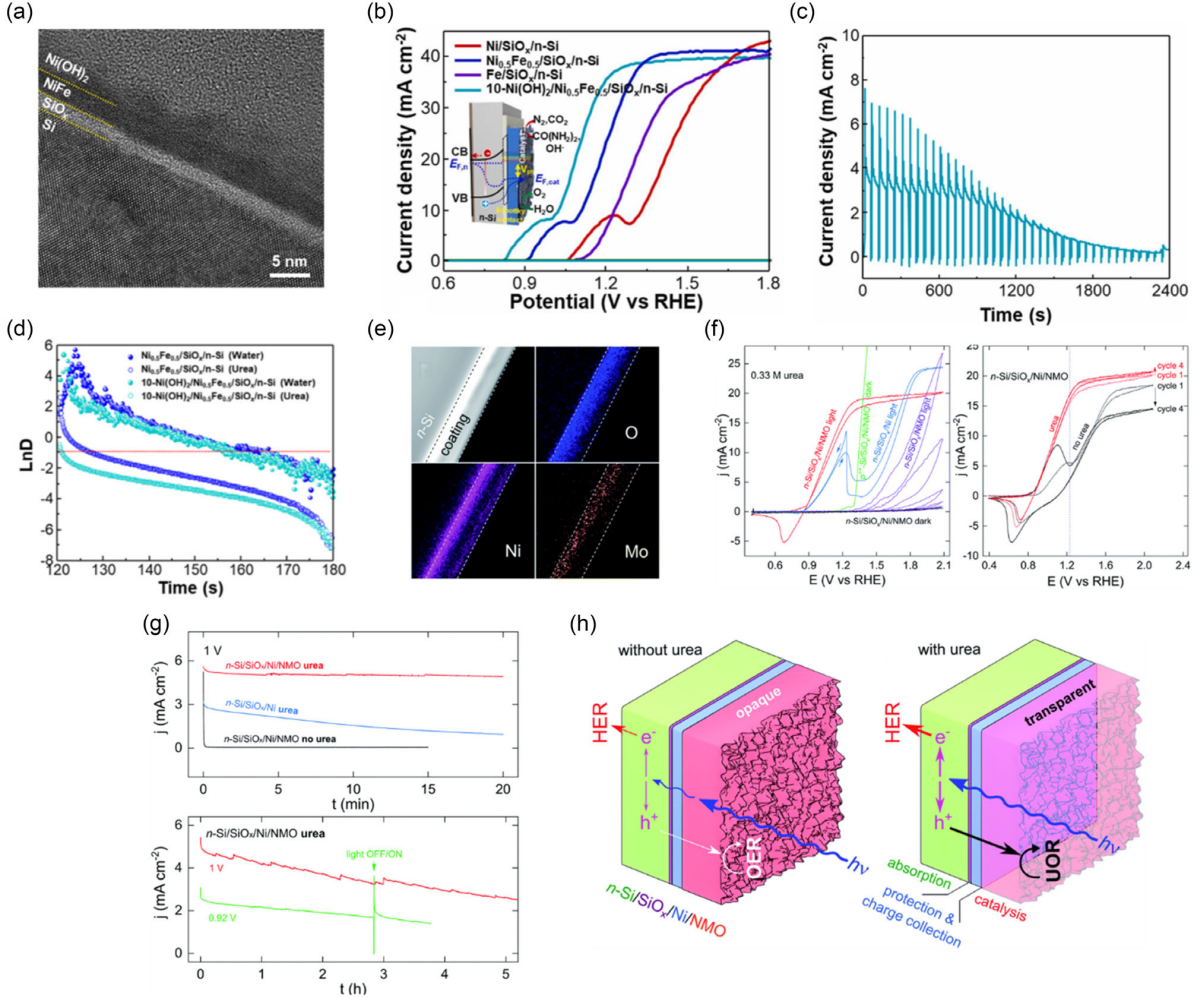
(a) TEM images of Ni(OH)_2_/Ni_0.5_Fe_0.5_/SiO_*x*_/n‐Si photoanodes, (b) LSV curves of n‐Si photoanode, (c) chronoamperometry (CA) curve of Ni(OH)_2_/Ni_0.5_Fe_0.5_/SiO_*x*_/n‐Si measured at 1 V versus RHE in the electrolyte of 0.33 M urea and 1 M NaOH solution under the chopped illumination, (d) transient decay measurements. Reproduced with permission [45]. Copyright 2022, Elsevier (e) STEM‐ADF and EDS‐STEM images of n‐Si/SiO_*x*_/Ni/NMO structure, showing elemental mapping of O (blue), Ni (purple), and Mo (orange). Dashed lines indicate the interface between the Ni and NMO layers, (f) cyclic voltammograms (CVs) recorded in the electrolyte of 1 M KOH and 0.33 M urea under dark and illuminated conditions. In the dark, n‐Si/SiO_*x*_/Ni/NMO (black curve) and p^++^‐Si/SiO_*x*_/Ni/NMO (green curve, forward scan). Under illumination: n‐Si/SiO_*x*_/NMO (purple curve, 5 successive cycles), n‐Si/SiO_*x*_/Ni (blue curve, arrows indicate the scan direction), and n‐Si/SiO_*x*_/Ni/NMO (red curve). (Left) 1st (thin line) and 4th (thick line) CVs recorded on illuminated n‐Si/SiO_*x*_/Ni/ NMO in 1 M KOH (black curves) and in 1 M KOH with 0.33 M urea (red curves). The standard potential of the O_2_/H_2_O redox couple (1.23 V vs. RHE) is indicated by a blue dotted line. Scan rate is 100 mV s^−1^. Illumination provided by a solar simulator (100 mW cm^−2^, AM 1.5G), (g) CA recorded at 1 V under illumination in 1 M KOH without urea on n‐Si/SiO_*x*_/Ni/NMO (black curve), and in 1 M KOH with 0.33 M urea on n‐Si/SiO_*x*_/Ni (blue curve), and n‐Si/SiO_*x*_/Ni/NMO (red curve). Additional CA on n‐Si/SiO_*x*_/Ni/NMO were recorded under illumination in 1 M KOH with 0.33 M urea at applied potentials of 1 V (red curve) and 0.92 V (green curve). (h) Schematics illustrating the photoanode structure and the effect of urea addition in the electrolyte on PEC performance adopted from [46]. Copyright 2022, the Royal Society of Chemistry.

Recent research shows that combining MIS photoanodes with suitable cocatalysts like nickel–iron layered double hydroxides or nickel phosphides can significantly improve charge transfer at interfaces and reduce the voltage needed for urea oxidation [45]. Lee et al. developed a heterostructure Ni(OH)_2_/Ni_0.5_Fe_0.5_/SiO_*x*_/n‐Si photoanode for PEC UOR, which includes a controlled native SiO_*x*_ layer (≈2 nm) beneath a 4 nm Ni_*X*_Fe_1‐*X*_ metallic layer, as shown in Figure 2a. This MIS device achieved a photovoltage of 530 mV. Adding a conformal Ni(OH)_2_ overlayer further improved charge transfer and stability, delivering a photocurrent density of 8.8 mA·cm^−2^ at just 0.83 V versus RHE under one‐sun illumination (Figure 2b). This enhancement is due to the hydrophilic Si/SiO_*x*_ interface, which suppresses surface state formation, raises the quasiFermi level, and promotes efficient, long‐term charge extraction. These results highlight the dual role of the Ni(OH)_2_ layer: facilitating interfacial charge transfer and promoting the initial PEC performance. Still, it may also introduce recombination pathways at the catalyst‐semiconductor interface, affecting stability over time. Stability tests (Figure 2c) showed a gradual photocurrent decline over 30 min, likely caused by increased interfacial recombination, indicated by early photocurrent overshoots. Transient decay measurements (Figure 2d) confirmed faster recombination kinetics during UOR than OER conditions. Interestingly, the Ni(OH)_2_ layer improved UOR activity, despite a 90 mV anodic shift compared to Ni_0.5_Fe_0.5_/SiO_*x*_/n‐Si, underscores the importance of optimizing catalyst composition and interface design to balance efficiency and stability in PEC UOR systems.

### 2.4 Amorphous and Optically Adaptive Ni—Mo—O Active Catalytic Layer for Selective PEC UOR

In PEC UOR systems, catalyst instability often causes unpredictable reaction pathways and lowers product selectivity. Therefore, integrating a strong and stable catalyst is crucial to ensure consistent reaction kinetics and dependable, selective product formation. Unfortunately, UOR electrocatalysts frequently undergo rapid deactivation, mainly due to poisoning of active sites by strongly adsorbed urea molecules and their reaction intermediates. During the six‐electron oxidation process, species such as NH_2_*, NH, CO*, and CN^−^ can build up on the catalyst surface, leading to the creation of carbonaceous or nitrogenous residues—including adsorbed CO, CN^−^, NO_*X*_^−^, or even polymeric nitrogen compounds—that act as insulating layers and block electron transfer at the interface. At the same time, competitive adsorption of electrolyte‐derived ions and changes to the catalyst surface can expose inactive sites or disrupt the original catalytic structure, speeding up performance decline. Overcoming these issues requires rational surface engineering to adjust intermediate binding energies, dynamic regeneration methods to remove blocking species, and catalyst designs that balance adsorption strength with turnover.

Dabboussi et al. created an MIS‐style Si photoanode by layering an amorphous Ni–Mo–O (NMO) film onto an n‐Si/SiO_*x*_/Ni junction. They started with Si(100) wafers and formed a 1.3–2 nm‐thick native SiO_*X*_ tunnel layer via chemical oxidation [46]. A 17 nm Ni layer was then deposited via DC magnetron sputtering to form the n‐Si/SiO_*x*_/Ni junction. Subsequently, Ni and Mo precursors were added through hydrothermal treatment, followed by arc annealing, which produced a 105 nm amorphous NMO layer, confirmed by [Scanning Transmission Electron Microscopy–Annular Dark‐Field (STEM‐ADF) and STEM‐based Energy‐Dispersive X‐ray Spectroscopy (STEM‐EDS) images (Figure 2e)]. Under one‐sun illumination, this Ni/NMO/SiO_*x*_/n‐Si photoanode achieved a 400 mV photovoltage and an onset potential of 0.9 V versus RHE for PEC UOR, maintaining high faradaic and photoconversion efficiencies over long periods (Figure 2f). In the presence of urea, the reaction occurs at relatively low potentials without fully oxidizing the Ni–Mo phase; instead, Ni cycles reversibly between Ni^2+^ and Ni^3+^ states, forming a more transparent NMO film that increases light absorption by the Si and boosts photocurrent. However, cyclic voltammetry showed a 96% decrease in photocurrent after five cycles due to photo—corrosion, emphasizing the protective role of the Ni layer. The pronounced cathodic peak at 1.2 V during reverse scans indicates in situ regeneration of active sites for PEC UOR. Chronoamperometry (Figure 2g) displays that after 5 h of continuous operation, the electrode retained about 50% of its initial photocurrent, with current spikes during illumination indicating transient hole accumulation at the interface. Figure 2h depicts the photoanode structure and the influence of urea in the electrolyte. Without urea, PEC OER occurs, and the NMO layer appears opaque, resulting in a low limiting photocurrent density. With urea, UOR is activated, making the NMO layer transparent and increasing the limiting photocurrent density. This study discloses the potential of MIS‐structured Si photoanodes with amorphous NMO overlayers for efficient PEC UOR by enabling effective charge transfer, optical transparency, and catalytic activity at low bias. Yet, the photocurrent degradation observed over cycles emphasizes the importance of achieving enhanced long‐term stability.

### 2.5 Ni‐Rich Catalytically Active Surfaces: Tuning Photovoltage via Junction Engineering

**FIGURE 3:**
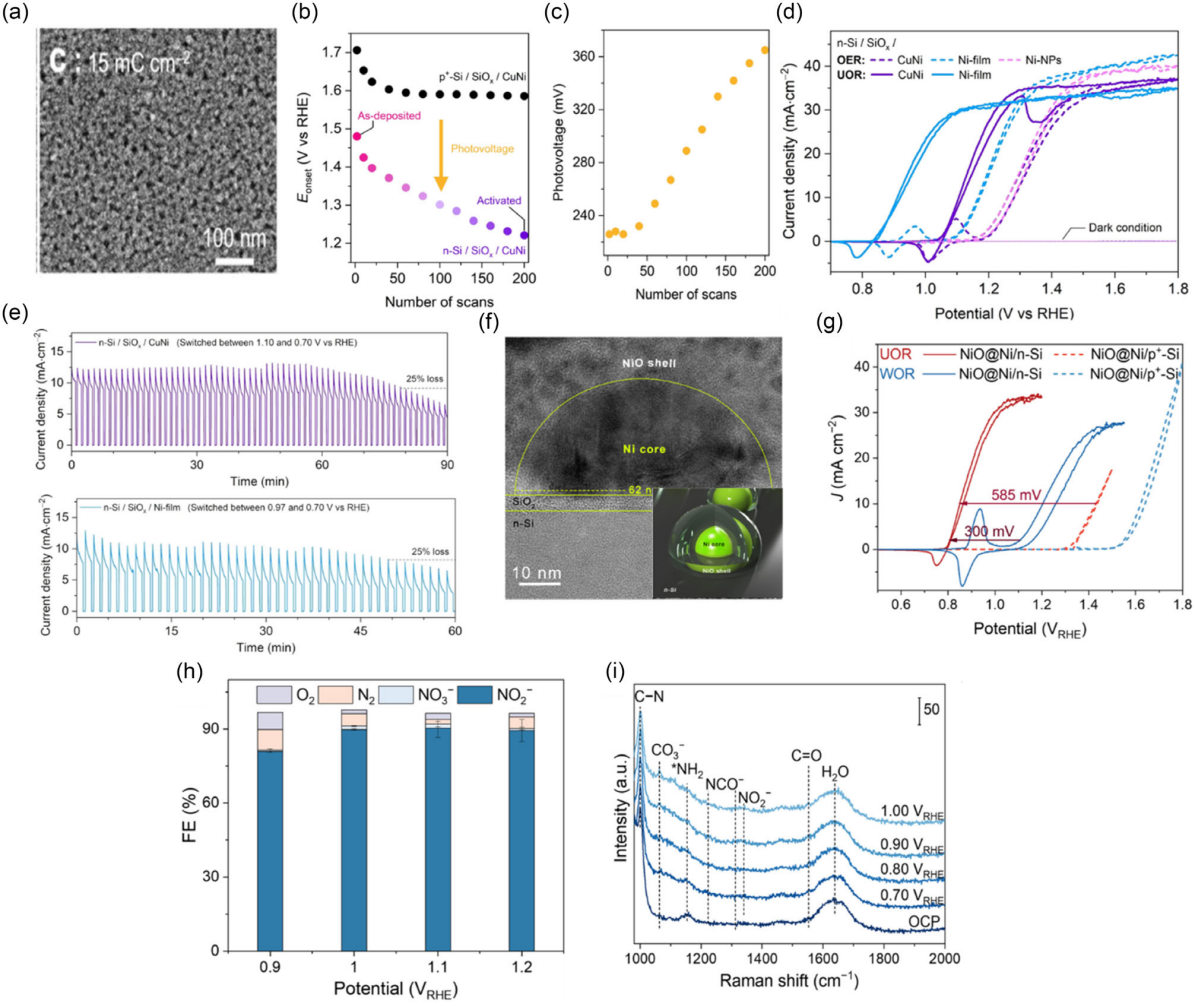
(a) FE‐SEM images of CuNi alloy for 15 mC cm^−2^, (b) onset potential (*E*_onset_) vs the number of CV cycles of n‐Si/SiO_*x*_/CuNi under 1‐sun illumination and p^+^‐Si/SiO_*x*_/CuNi in the dark, tested in 1 M KOH, (c) photovoltage versus the number of CV cycles of n‐Si/SiO_*x*_/CuNi, (d) CVs of activated n‐Si/SiO_*x*_/CuNi photoanodes prepared with different electrochemical deposition in 1 M KOH containing 0.33 M urea, (e) CA curves of n‐Si/SiO_*x*_/CuNi and n‐Si/SiO_*x*_/Ni‐film for UOR in the electrolyte of 1 M KOH with 0.33 M urea under 1‐sun illumination, Reproduced with permission [47]. Copyright 2024, Wiley‐VCH (f) HR‐TEM images of NiO@Ni/n‐Si photoanode, (g) CV profiles of NiO@Ni/n‐Si (under front illumination of AM 1.5 G, 100 mW cm^−2^), (h) products distribution of PEC UOR using NiO@Ni/n‐Si photoanode under various potentials, and (i) potential‐dependent Raman spectra of surface‐bound intermediates during the PEC UOR on NiO@Ni/n‐Si in 1 M NaOH containing 0.33 M urea, Reproduced with permission [48]. Copyright 2025, Wiley‐VCH.

CuNi alloys are highly active, poison‐resistant catalysts for UOR. In one study, a CuNi film was electrodeposited onto Si photoanodes at –1.2 V versus SCE, with thickness and uniformity carefully controlled by charge density instead of deposition time (seeing Figure 3a). Monitoring the onset potential (*E*_onset_) over successive scans revealed different behaviors between p^+^‐Si and n‐Si substrates (Figure 3b): For p^+^‐Si/SiO_*x*_/CuNi, *E*_onset_ decreased in the first 50 cycles before stabilizing. In contrast, n‐Si/SiO_*x*_/CuNi showed a consistent cathodic shift of E_onset_ over 200 cycles. Photovoltage—defined as the *E*_onset_ difference at 5 mA·cm^−2^ between illuminated n‐Si/SiO_*x*_/CuNi and dark p^+^‐Si/SiO_*x*_/CuNi—increased from 226 mV after 50 cycles to 365 mV after 200 cycles (seeing Figure 3c). Under one‐sun illumination, the activated n‐Si photoanodes, modified with various cocatalysts, were tested with and without urea (Figure 3d). In urea‐containing electrolyte, the n‐Si/SiO_*x*_/CuNi electrode displayed a notable cathodic shift in E_onset_—indicating a lower thermodynamic barrier for UOR compared to OER—and a sharp increase in current density coinciding with the Ni^3+^/Ni^2+^ redox couple, suggesting in situ NiOOH formation. Ultimately, the CuNi‐modified n‐Si photoanode achieved UOR at an onset potential of 1.05 V versus RHE and reached 31 mA·cm^−2^ at 1.23 V versus RHE. This modification significantly enhanced photocurrent, improved charge separation, and reduced recombination, offering strong dual‐function PEC activity for both water and urea oxidation with outstanding long‐term stability (Figure 3e). These results demonstrate that bimetallic alloy cocatalysts offer a promising approach to achieve both high catalytic activity and resistance to poisoning and deactivation [47]. To advance toward practical applications, future research should focus on optimizing alloy composition, engineering stable semiconductor–cocatalyst interfaces, and improving long‐term photostability—key steps for developing scalable, durable PEC systems capable of efficient solar hydrogen production and wastewater treatment.

Overall, the above used catalyst for PEC UOR underscores how alloying and interfacial engineering strategically influence efficiency, stability, and cost with the performance of MIS–Si photoanodes being highly sensitive to cocatalyst composition. NiFe alloys (particularly Ni_0.5_Fe_0.5_) exhibit excellent activity, with low onset potentials (≈1.02 V vs. RHE), high photovoltage (≈530 mV), and >90% charge injection efficiency. However, their stability is limited due to Fe dissolution in alkaline media, often leading to degradation within a few hours. Similarly, Ni decoration with Ni(OH)_2_ improves photocurrent density (41 mA cm^−2^) and fill factor (25.7%), but suffers from poor durability in strong alkaline electrolytes. By contrast, Ni–Mo–O (NMO) layers on Si/SiO_*x*_/Ni junctions demonstrate unique advantages associated with their amorphous character and optical versatility. These films deliver a photovoltage of ≈400 mV and onset potentials of 0.87–0.9 V, along with high photocurrents under urea oxidation (17.3 mA cm^−2^ at 1.23 V vs. RHE). They also operate stably for several hours at low overpotentials, with faradaic efficiencies up to 85%. Although Mo leaching remains a concern, it simultaneously increases NiOOH active site density, partially mitigating performance decay. The enhanced light transmission enabled by this structure further distinguishes it from other cocatalysts.

CuNi alloys, prepared via electrodeposition, show low onset potentials (1.05 V for UOR, 1.15 V for OER) and high photocurrent densities (31 mA cm^−2^ at 1.23 V). Importantly, these alloys resist poisoning more effectively than pure Ni and exhibit improved operational stability. Upon activation, partial Cu dissolution produces NiO_*x*_‐rich surfaces, which increase barrier height and photovoltage. Compared with planar Ni films, CuNi alloys achieve slightly lower peak activity but offer superior durability, along with low‐cost and scalable fabrication advantages through electrodeposition.

In summary, NiFe demonstrates outstanding efficiency but lacks stability; NiMoO balances moderate stability with optical adaptability; and CuNi provides a practical trade‐off between activity, durability, and cost‐effectiveness. Together, these examples highlight the critical role of interface engineering and alloying in advancing MIS–Si photoanodes, and illustrate the broader design principles and trade‐offs that guide cocatalyst selection for PEC applications.

### 2.6 Ni‐Based Photoanodes: Catalytic Pathways and Energetics of C–N Bond Activation

A recent study explored how adsorbate–adsorbate interactions on semiconductor surfaces can be harnessed to overcome kinetic limitations and enhance efficiency and selectivity in urea oxidation [48]. The stable resonance structure and strong C—N bonds lead to slow reaction kinetics during UOR, particularly in PEC systems. Effective C—N activation directs the reaction toward complete oxidation to CO_2_ and N_2_, preventing undesired partial‐oxidation by‐products such as NO_2_^−^.

Dang et al. reported the intrinsically slow C—N bond cleavage in urea by employing high‐valent metal‐oxo species and enhancing specific adsorbate–adsorbate interactions. They developed a NiO@Ni/n‐Si photoanode via a two‐step electrochemical deposition: first, metallic Ni nanoparticles (≈62 nm) were electrodeposited onto a 4 nm SiO_2_–passivated n‐Si wafer, creating a protective Schottky‐junction layer. Then, a conformal 16 nm NiO shell was grown around the Ni cores, forming a distinct core–shell structure, confirmed by HR‐TEM images (Figure 3f). Under AM 1.5 G illumination, the electrode reached a peak photocurrent density of 30.5 mA cm^−2^ at 1.00 V versus RHE (Figure 3g). In a urea‐containing electrolyte, Faradaic efficiencies for NO_2_^−^ of about 80% were achieved at 0.90 V versus RHE, increasing to 90% between 1.00 and 1.20 V versus RHE, outperforming most previous UOR systems (Figure 3h). The high selectivity for nitrite indicates the formation of the favorable N—O bond, facilitated by the NiO shell during oxidation. Operando Raman spectroscopy (Figure 3i) revealed new vibrational bands in the 1000–1100 cm^−1^ range, linked to C–N stretching modes or urea fragments, providing direct evidence of enhanced C—N bond activation during the process. Mechanistic studies show that surface Ni(IV)=O species strongly polarize adsorbed urea molecules, promoting N—O bond formation while weakening the C—N bond. As Ni(IV)=O coverage increases, the UOR activation energy decreases from 0.74 to 0.41 eV, boosting the reaction rate by over 100 times. These results emphasize the role of high‐valent metal–oxo species in overcoming the C—N bond cleavage barrier in PEC UOR. Future efforts should focus on controlling metal–oxo species and adsorbate interactions to develop more efficient, selective photoanodes for solar‐driven urea conversion and wastewater treatment.

## 3 PEC Ammonia Splitting (PEC AOR)

Ammonia (NH_3_) is both a widespread environmental pollutant and a promising hydrogen carrier: it contains 17.6 wt% hydrogen, is easily liquefiable under moderate conditions, and can utilize existing fuel‐distribution infrastructure. NH_3_ can be converted to high‐purity H_2_ and harmless N_2_ with minimal by‐products through catalytic decomposition or selective oxidation, usually using Ru, Pt, or Ni‐based catalysts [49, 50]. These reactions are performed at elevated temperatures (600–800°C) and pressures (410–1000 kPa) to ensure practical conversion rates. However, reliance on costly noble metals and the possible formation of nitrous oxide (N_2_O)—a potent greenhouse gas—pose ongoing challenges for catalyst design and process optimization. To overcome the limitations of thermocatalytic methods and improve energy and resource efficiency, PEC oxidation of ammonia offers a low‐temperature, potentially solar‐driven pathway. This approach reduces operational costs and boosts system efficiency by coupling fuel production with valuable chemical transformations [15, 51]. Moreover, the PEC approach for ammonia oxidation produces hydrogen at rates approximately 2–2.7 times higher than the stoichiometric hydrogen demand of the Haber–Bosch process; this surplus hydrogen significantly lessens reliance on external H_2_ feedstock‐the primary cost factor in conventional ammonia synthesis‐and enhances the overall economic viability of the process. Aside from increasing hydrogen yield and decreasing dependance on expensive feedstocks, PEC ammonia oxidation offers broader technological and environmental benefits. Its integration into sustainable energy and chemical systems provides unique advantages that surpass traditional thermocatalytic methods [52].

PEC ammonia oxidation (Equation 15) is significant because it:

(1) Replacing water oxidation with ammonia oxidation at the photoanode lowers the energetic barrier, accelerates interfacial kinetics, and improves overall energy efficiency.

(15) $$2{\mathrm{NH}}_{3}\to N_{2}+6H^{+}+6e^{-}(E^{0}=0.06V\mathrm{vs}.\mathrm{RHE})$$

Additionally, this process lowers the thermodynamic potential by over 1V, enhances the number of electrons transferred per molecule, and accelerates interfacial charge transfer, collectively allowing hydrogen production at the cathode under milder and more sustainable conditions. (2) PEC ammonia oxidation removes toxic ammonia from wastewater by converting it into harmless N_2_ and H_2_ using sunlight, contributing to sustainable hydrogen generation without high‐energy inputs. (3) Combining PEC ammonia oxidation with renewable ammonia sources offers a scalable approach for localized hydrogen production, helping to decarbonize energy use and infrastructure logistics. (4) The demand for visible‐light absorption, effective charge separation, corrosion resistance, and ammonia‐selective catalysis drives innovation in photoanode materials (e.g., BiVO_4_, WO_3_, α‐Fe_2_O_3_), surface engineering, and metal‐oxo active site design. These developments not only enhance PEC NH_3_ oxidation performance but also pave the way for more sustainable and efficient solar‐to‐chemical conversion systems [53, 54].

Understanding the mechanistic complexity of ammonia oxidation is crucial, as the reaction pathway and resulting nitrogen products are highly sensitive to factors such as catalyst composition, applied potential, pH, and reaction environment. In general, NH_3_ can be oxidized via multiple reaction pathways, yielding different nitrogen products depending on conditions. The overall reactions can produce primarily molecular N_2_, nitrous oxide (N_2_O), or nitric oxide (NO). For example, under stoichiometric conditions, a “complete” oxidation of ammonia proceeds via the following (Equation 16):

(16) $$2{\mathrm{NH}}_{3}+1.5O_{2}\to N_{2}+3H_{2}O$$

forming molecular N_2_ as the primary product.

However, under more oxygen‐rich conditions, partial oxidation pathways can dominate, leading to the formation of undesired byproducts such as nitrous oxide (N_2_O) or nitric oxide (NO). A representative reaction (Equation 17) is:

(17) $$2{\mathrm{NH}}_{3}+2.5O_{2}\to 2\mathrm{NO}+3H_{2}O$$

Looking ahead, the ability to precisely control reaction conditions and customize catalyst selectivity will be essential for steering ammonia oxidation toward environmentally friendly products like N_2_, while reducing the formation of unwanted byproducts such as NO and N_2_O. Improving these control strategies offers great potential for enabling cleaner nitrogen‐cycle technologies and sustainable chemical manufacturing.

### 3.1 Mechanistic Insights and Selectivity Control

**FIGURE 4:**
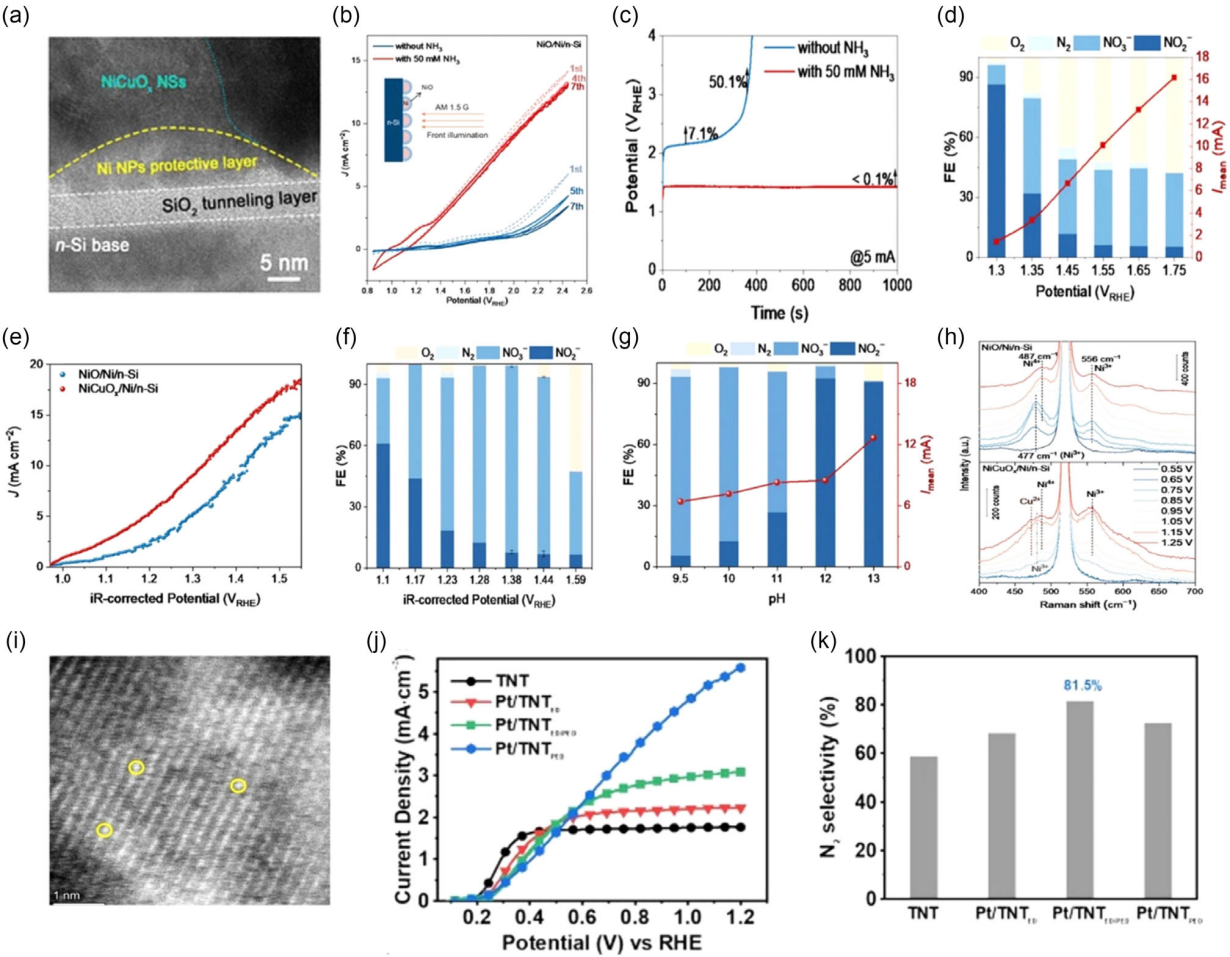
(a) Cross‐sectional HR‐TEM of the NiCuO_*X*_/Ni/n‐Si photoanode, (b) CV curves of the NiO/Ni/n‐Si photoanode in 0.1 M NaClO_4_ with or without 50 mM NH_3_ (pH =  11), (c) CP curves of the NiO/Ni/n‐Si photoanode in 0.1 M NaClO_4_ with or without 50 mM NH_3_ (pH =  11) under a constant photocurrent of 5 mA, (d) potential‐dependent FE of products and the corresponding mean photocurrent as a function of the applied potential for PEC electrolysis using a NiO/Ni/n‐Si photoanode in 0.1 M NaClO_4_ with 100 mM NH_3_, (e) J–V curves of NiCuO_*X*_/Ni/n‐Si and NiO/Ni/n‐Si photoanodes with iR‐correction tested in 0.2 M NaClO_4_ with 150 mM NH_3_ under AM 1.5 G illumination, (f) Potential‐dependent product distribution, (g) pH‐dependent product distribution of AOR on the NiCuO_*X*_/Ni/n‐Si photoanode, measured at 1.40 *V*_RHE_ in 0.1 M NaClO_4_ electrolyte (adjusted to pH 13 using 0.1 M NaOH), with 100 mM NH_3_, (h) Operando potential‐dependent Raman spectra of the NiO/Ni/n‐Si and NiCuO_*X*_/Ni/n‐Si photoanodes during the PEC AOR. Adopted from [55] Copyright 2022, the Royal Society of Chemistry, (i) HAADF–STEM images of Pt/TNT_ED_, (j) photocurrent density‐potential curves of different catalysts for PEC UOR and (k) PEC N_2_ selectivity of the obtained catalysts for UOR, measured under a 0.7 *V*_RHE_ bias and UV irradiation over 120 min. Reproduced with permission [56]. Copyright 2025, Elsevier.

Dang et al. created a NiCuO_*X*_/Ni/n‐Si photoanode by electrochemically depositing Ni–Cu bimetallic nanosheets onto a SiO_*X*_‐passivated n‐Si wafer (seeing Figure 4a). This two‐step process involves first depositing a thin Ni film to act as a protective Schottky‐junction layer [55], followed by growing Ni–Cu oxide nanosheets just above, which function as the catalytically active overlayer. Under AM 1.5 G illumination (100 mW·cm^−2^), the optimized NiCuO_*X*_/Ni/n‐Si photoelectrode achieved a partial photocurrent density of approximately 12 mA·cm^−2^ at a low applied potential of 1.38 V versus RHE, with a maximum faradaic efficiency (FE) of 99% for NO_*X*_ production (seeing Figure 4b). Chronopotentiometry (CP) curves (seeing Figure 4c) indicated that NH_3_ significantly stabilizes the photoanode. In an electrolyte containing NH_3_, a steady current density of 5 mA·cm^−2^ was maintained at 1.42 V versus RHE for 1000 s with negligible potential drift (<0.1%).

In contrast, the same current required a much higher potential of 2.01 V versus RHE without NH_3_. Product analysis (Figure 4d) confirmed the co‐oxidation of NH_3_ and H_2_O, generating soluble NO_*X*_ species and molecular O_2_. At 1.30 *V*_RHE_, the FE for NO_2_^−^ reached 86%, indicating highly selective partial oxidation. As the potential increased, a gradual conversion of NO_2_^−^ to NO_3_^−^ was observed, consistent with stepwise nitrogen oxidation. Comparative J–V curves (Figure 4e) showed that the NiCuO_*X*_/Ni/n‐Si photoanode had higher photocurrent density and stability than the NiO/Ni/n‐Si, due to its ability to produce NO_*X*_ selectively. To better understand the active species’ evolution under applied bias and the origins of high performance and selectivity in NO_*X*_ formation, Operando Raman spectroscopy (Figure 4h) was performed under operational conditions. At potentials as low as around 0.65 *V*_RHE_, NiO oxidized to NiOOH, shown by vibrational modes at ≈477 cm^−1^ (*E*_g_) and 556 cm^−1^ (A_1g_). When the potential exceeded 0.95 *V*_RHE_, a new band at 487 cm^−1^ appeared, indicating the formation of Ni^4+^–O species. These high‐valent Ni centers are crucial for efficient NH_3_ oxidation, promoting both high activity and selectivity toward products.

Furthermore, Zhang et al. prepared self‐organized anatase TiO_2_ nanotube (TNT) arrays on Ti foil through anodization and then electrochemically deposited atomically dispersed Pt onto the TNT surface [56]. High‐angle annular dark‐field STEM (Figure 4i) clearly shows isolated Pt atoms evenly anchored on the nanotubes. Under UV illumination (365 nm, 15 W) in 1 M KOH with 0.1 M NH_4_OH (pH 13), the Pt‐decorated TNT photoanode shows a significant increase in anodic current density above 0.5 V_RHE_, compared to bare TNT (Figure 4j). This increase is due to strong Pt–TiO_2_ electronic coupling within the nanotube structure, which helps charge transfer. This improved charge transfer efficiency directly influences the reaction pathway, allowing for more selective nitrogen evolution during ammonia oxidation. Ammonia oxidation on this photoelectrode produces molecular N_2_ at a rate nearly half of the NH_3_ consumed, indicating high N_2_ selectivity toward N_2_ formation. Gas analysis confirms that the Pt/TNT photoanode achieves an N_2_ selectivity of about 81.5% under operational conditions (Figure 4k). This outstanding performance results from the incorporation of single‐atom Pt, which greatly reduces interfacial charge transfer resistance, thus enhancing ammonia oxidation kinetics and promoting the preferred formation of N_2_ over undesired byproducts.

**FIGURE 5:**
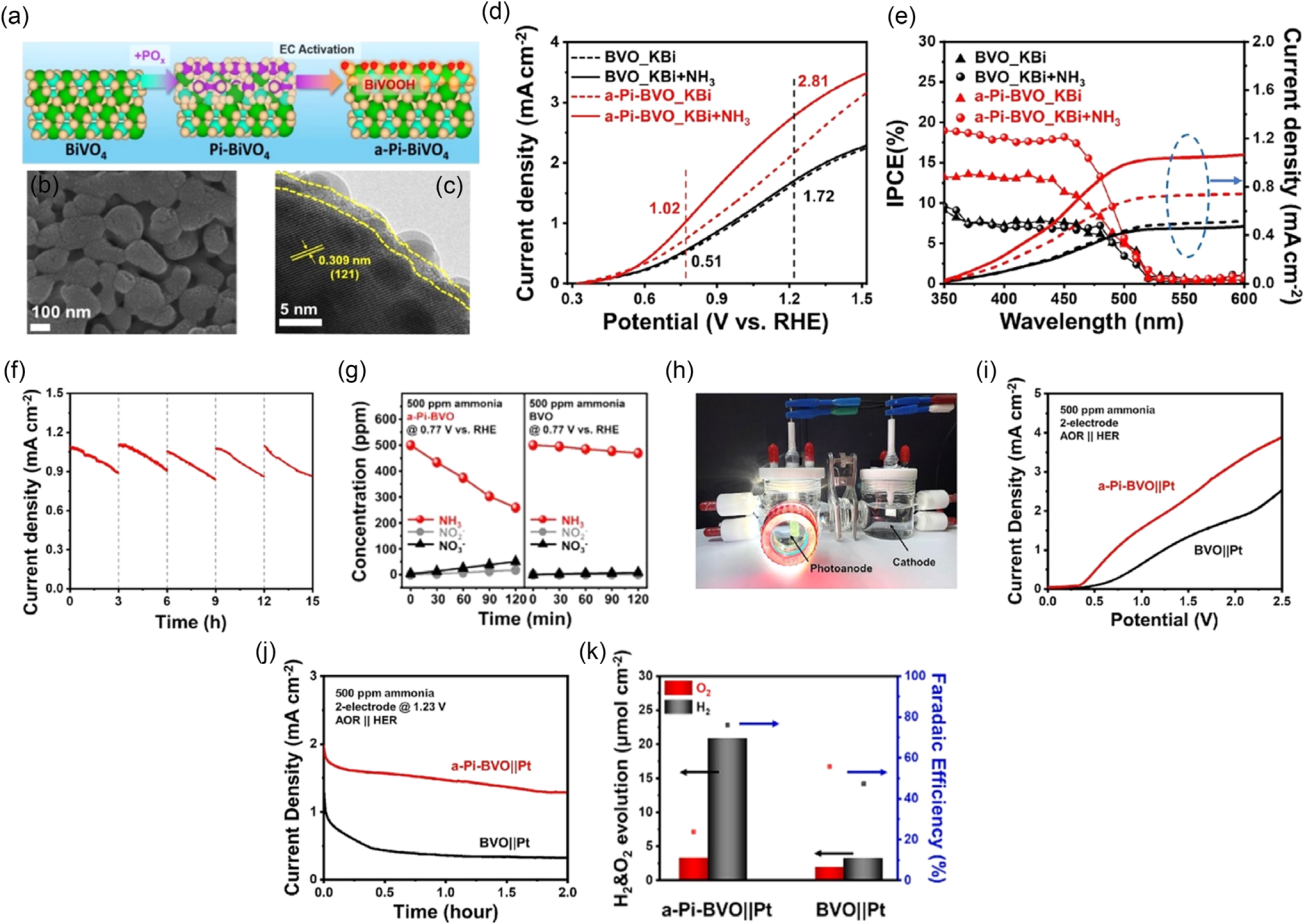
(a) Schematic illustration on the preparation process of a‐Pi‐BVO photoanode, (b) SEM images of a‐Pi‐BVO photoanodes, (c) TEM images of a‐Pi‐BVO photoanodes, (d) LSV curves under illumination and under dark condition, (e) IPCE of different photoanodes measured at 0.77 V versus RHE in 1 M KBi with and without 500 ppm NH_3_ and integrated photocurrent densities from the corresponding IPCE spectra, (f) cyclic stability test of a‐Pi‐BVO in the 1 M KBi + 500 ppm NH_3_ at 0.77 V versus RHE, (g) concentration distribution of ammonia, nitrate and nitrite in the PEC AOR under pristine BVO and a‐Pi‐BVO photoanode, (h) photograph of two‐electrode configuration for PEC ammonia electrolysis system using a‐Pi‐BVO as photoanode and Pt plate as cathode, H_2_ gas bubbles observed on the Pt electrode, (i) LSV curves of a‐Pi‐BVO||Pt and BVO||Pt cell, (j) CA test of a‐Pi‐BVO||Pt and BVO||Pt cell at a cell voltage of 1.23 V in 1 M KBi electrolyte with 500 ppm ammonia under 1 sun illumination, and (k) H_2_ and O_2_ evolution of a‐Pi‐BVO||Pt and BVO||Pt cells after 60 min of reaction. Reproduced with permission [56]. Copyright 2025, Elsevier.

Another study identified a precisely engineered phosphate‐derived bismuth‐vanadium oxyhydroxide (BiVOOH) overlay on BiVO_4_ photoanodes, which significantly boosts PEC‐ AOR and downstream hydrogen production in dilute alkaline solutions [57]. A simple three‐step process—fabricating BiVO_4_, vapor‐phase phosphorylation (Pi‐BVO), and long‐term electrochemical activation—creates a 3–4 nm thick bimetallic BiVOOH shell. SEM/TEM images (Figures 5b–c) reveal the surface change from smooth in BVO to rough, worm‐like in Pi‐BVO, and even rougher with an ≈3 nm coating in a‐Pi‐BVO. The BiVOOH layer extracts photogenerated holes from the BiVO_4_ bulk and supplies high‐valence Bi and V sites with —OH groups, enhancing six‐electron AOR pathways. Under AM 1.5 G illumination in 500 ppm NH_3_ at pH 10, a‐Pi‐BVO reaches a photocurrent density of 1.02 mA·cm^−2^ at 0.77 V versus RHE—ten times that of pristine BVO (0.10 mA·cm^−2^) and much higher than Pi‐BVO (Figure 5d). Incident photon‐to‐current efficiency (IPCE) measurements show increased activity below 500 nm only on a‐Pi‐BVO, aligning with AOR‐specific activity (Figure 5e). Cyclic stability tests of the a‐Pi‐BVO photoanode, with electrolyte refreshed every 3 h (Figure 5f), show the photocurrent gradually decreasing within each cycle due to NH_3_ depletion, but nearly recovering after refreshing. Figures 5g shows UV–vis measurements of NH_3_, NO_2_^−^, and NO_3_^−^ after 2 h of PEC AOR at 0. 77 V versus RHE, indicating enhanced PEC AOR activity due to the metal‐oxyhydroxide layer: pristine BVO yielded 31 ppm NH_3_ with ≈67.4% N_2_ selectivity, whereas a‐Pi‐BVO produced 241 ppm NH_3_ with 71.4% N_2_ selectivity, confirming surface modification primarily improved PEC UOR. In a two‐electrode PEC cell (a‐Pi‐BVO||Pt), only 1 23 V was necessary for concurrent AOR at the photoanode and H_2_ evolution at the Pt cathode. The photocurrent reached 1.91 mA·cm^−2^—1.9 times higher than unmodified BVO||Pt‐ and H_2_ production over an hour was 6.5 times greater, with an O_2_:H_2_ ratio of 1:6, confirming the dominance of AOR over OER. The FE for oxygen dropped to 23.7% from 55.7% (on BVO), further supporting AOR selectivity (Figure 5h‐k).

These results clearly demonstrate that a‐Pi‐BVO significantly enhances PEC UOR by increasing photocurrent, nitrogen conversion, and hydrogen generation, while maintaining AOR selectivity over OER. The recoverable performance after electrolyte refresh also indicates good operational stability. Moving forward, further optimization of surface composition, long‐term durability under continuous operation, and extension to real wastewater conditions will be essential to advance a‐Pi‐BVO and related systems toward practical PEC ammonia remediation and fuel production applications.

## 4 PEC NO Oxidation to NO_3_^−^

Nitric oxide (NO) is a significant air pollutant released from fossil fuel burning and human activities. These emissions contribute to issues like acid rain, photochemical smog, PM2.5 formation, ozone layer depletion, and climate change, in addition to causing health problems related to respiration and the cardiovascular system. Traditional NO_*X*_ removal methods, such as adsorption, nonselective catalytic reduction, and ammonia‐based SCR, are mainly effective at high temperatures and concentrations, like in power plant exhaust. However, they become less efficient or more expensive at lower concentrations typical of ambient or indoor air. For example, SCR systems operate at 300°C–400°C with added ammonia, which is impractical and can generate secondary waste such as N_2_O or NH_4_NO_3_ aerosols. Photo‐driven oxidation of NO_*X*_ offers a greener, cost‐effective solution, especially for trace amounts at room temperature. This process uses photoactive semiconductor materials that absorb light and generate electron–hole pairs, producing reactive oxygen species (OH,·O_2_^−^, ^1^O_2_) to oxidize NO into nitrate (NO_3_^−^). It operates under ambient conditions without extra chemicals, directly converting NO_*X*_ into harmless or manageable forms. Nitrates can be washed away or captured in water, preventing gaseous NO_*X*_ emissions and avoiding toxic byproducts like NO_2_ that partial reactions might produce. The typical pathway involves NO converting to NO_2_ and then to NO_3_^−^, mediated by photogenerated holes and radicals (e.g., ·OH). Most NO ends up as stable nitrate.

Briefly, the PEC NO_*X*_ oxidation proceeds through multiple pathways governed by reactive oxygen species (ROS) and interfacial conditions. Hydroxyl radicals (•OH), generated by photogenerated holes, drive deep oxidation via the sequence NO → HONO → NO_2_ → NO_3_^−^, with humidity strongly influencing hydroxylation and HONO release. In contrast, conduction band reduction of O_2_ yields superoxide (•O_2_^−^), which reacts rapidly with NO to form peroxynitrite (ONOO^−^) that subsequently converts to nitrate. This pathway is highly efficient under moderate humidity and enhances nitrate selectivity while suppressing NO_2_ evolution. Singlet oxygen (^1^O_2_) contributes only marginally, acting mainly on nitrite/nitrate intermediates rather than NO directly [58, 59, 60, 61, 62, 63, 64, 65, 66]. In photocatalytic systems, recombination of electrons and holes limits active species for surface reactions. Moreover, these setups lack external bias control, which hampers their ability to maintain optimal reaction potentials, reducing selectivity and efficiency. PEC NO oxidation systems address these issues by providing external bias to improve charge separation, stabilize reactive intermediates, and enhance reaction kinetics.

The environmental motivation for PEC NO oxidation is its ability to remove NO_*X*_ more effectively, especially at low concentrations and room temperature, surpassing traditional and purely photocatalytic methods. This enhances air purification in indoor spaces and urban areas, where current solutions often underperform. In a PEC reactor, NO is oxidized on the photoanode's surface, and the resulting nitrate dissolves or rinses into an aqueous electrolyte. This process both cleans the air and captures nitrate in water for potential reuse or treatment. Moreover, photogenerated holes in the illuminated photoanode stay available to facilitate more efficient NO oxidation. The NO oxidation product, NO_3_^−^, typically remains on the photoanode surface or in the electrolyte and can potentially be used as fertilizer. NO oxidation aligns well with solar‐driven chemistry because the redox energetics match semiconductor bandgaps. Since NO has a lower bond dissociation energy than N_2_, it is easier to oxidize NO into nitrate than to break triple bond of N_2_, as in nitrogen fixation. This thermodynamic advantage indicates that sunlight of suitable wavelengths provides enough energy to drive NO to NO_3_^−^ conversion on a properly biased photoanode. This process is straightforward and cleaner than conventional thermal methods. Using NO, a waste product, as a starting material for nitrate production is chemically efficient, bypassing energy‐intensive Haber‐Bosch and Ostwald processes. Consequently, PEC NO oxidation is driven by the fact that using NO as a feedstock consumes less energy compared to producing nitrates from N_2_. Recent research on PEC NO oxidation to nitrate focuses on system configurations that ensure efficient, selective conversion.

### 4.1 Hydroxyl Radical‐Driven Photoelectrocatalytic NO Oxidation in a Flow‐Type Photoanode Reactor

**FIGURE 6:**
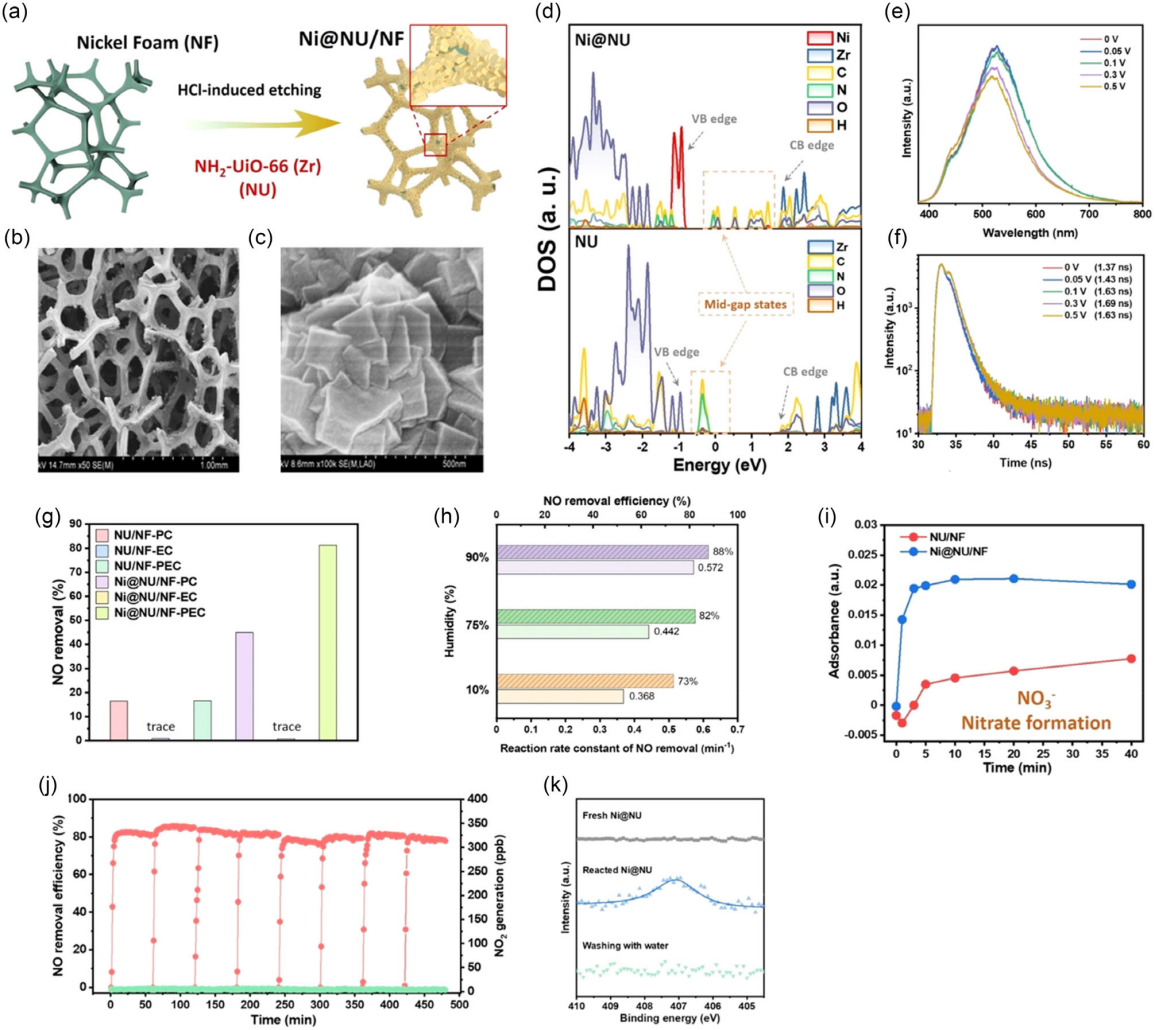
(a) Schematic illustrations of the fabrication process for Ni@NU/NF photoanodes, (b) FE‐SEM images of Nickel foam, (c) FE‐SEM images of Ni@NU/NF, (d) DOS plots for NU and Ni@NU, (e) the in situ steady‐state PL spectra of Ni@NU/NF at different voltages, (f) corresponding time‐resolved PL spectra of Ni@NU/NF at different voltages, (g) NO removal performance of NU/NF and Ni@NU/NF under PC, EC and PEC conditions, (h) NO removal performance of Ni@NU/NF under different humidity, (i) in situ DRIFTS spectra of the NO adsorption for nitrate (1258 cm^−1^), (j) long‐term PEC NO removal over Ni@NU/NF and (k) N1*s* XPS of the surface nitrogen species on Ni@NU/NF before and after washing. Reproduced wiht permission. [67] Copyright 2023, Wiley‐VCH.

This study addresses the environmental challenge of removing dilute nitrogen monoxide (NO), a major air pollutant from sources like vehicle exhaust and industrial emissions, while converting it into useful nitrate for sustainable agriculture. Traditional methods like SCR are energy‐intensive and less effective at low NO concentrations (ppb range). Photocatalytic oxidation offers greener solutions but often suffers from low selectivity due to reactive oxygen species (ROS) that can convert NO into unwanted NO_2_. To improve this, the authors developed a PEC system that directs ROS mainly to produce hydroxyl radicals (·OH), which selectively oxidize NO fully into nitrate. The key innovation is a nickel‐modified NH_2_‐UiO‐66 (Zr) (briefly marked as NU) MOF coated on 3D nickel foam (Figure 6a) [67]. The nickel foam provides a hierarchical mesoporous structure with a surface area of 420 m^2^·g^−1^ (average pore size 2.58nm), aiding gas diffusion and enabling solid nitrate storage—preventing catalytic site blockage and byproduct formation. FE‐SEM images reveal that the Ni foam surface is densely covered with irregular polyhedra (Figure 6b–c). Besides its structural advantages, nickel also enhances PEC activity by increasing ·OH production via water oxidation at the photoanode and by improving electron–hole separation efficiency under visible light and low bias (0.3 V).

DFT calculations (Figure 6d) show that the energy barrier for water oxidation to ·OH is significantly lower with Ni addition (−2.34eV for Ni@NU vs. −0.52eV for unmodified NU), indicating that water splits more easily into ·OH radicals on the Ni@NU/NF surface, boosting NO oxidation. Additionally, DFT results suggest Ni doping introduces midgap states, slightly narrows the bandgap, and forms a Schottky barrier at the Ni@NU/NF interface, enhancing charge separation. These theoretical findings are supported by experimental data showing higher photocurrent, reduced charge–transfer resistance, and longer lifetimes of photogenerated carriers under bias (Figure 6e–f). An optimal bias of 0.3V was identified, maintaining electron flow to the cathode and hole accumulation at the anode, promoting continuous ·OH production. Under this condition, NO removal efficiencies within 10 min were 16% for photocatalysis (PC), 1% for electrocatalysis (EC), and 17% for photoelectrocatalysis (PEC) using pure NU/NF (Figure 6i). The influence of humidity on NO oxidation was also examined (Figure 6j). As expected, PEC performance on Ni@NU/NF was significantly enhanced, showing higher removal efficiency and reaction rate constant, emphasizing the crucial role of ·OH radicals in NO oxidation.

X‐ray photoelectron spectroscopy (XPS) analysis further supported these findings, showing stronger nitrate signals on Ni@NU/NF compared to pristine NU/NF (Figure 6k), which correlates with improved NO removal performance. Nitrate formation displayed a linear relationship with ·OH consumption, confirming that ·OH is the primary oxidant converting NO to nitrate. Moreover, Ni@NU/NF demonstrated good stability over multiple PEC cycles and limited NO_2_ formation during continuous operation (Figure 6j). Long‐term cycling tests confirmed steady NO removal and minimal NO_2_ emissions. The nitrate could be effectively washed off and recovered at about 90% yield, indicating the feasibility of the reactor for both air purification and nitrogen recycling into fertilizers. Its durable 3D mesoporous structure ensured high storage capacity and minimized catalyst deactivation. To quantify nitrate formation on Ni@NU/NF, nitrate was washed off and measured via ion chromatography. Postwashing (Figure 6m), no nitrogen was detected on fresh Ni@NU/NF, confirming complete nitrate recovery and catalyst reusability for long‐term use.

### 4.2 Significance of Gas‐Phase PEC Reactor for NO Oxidation

**FIGURE 7:**
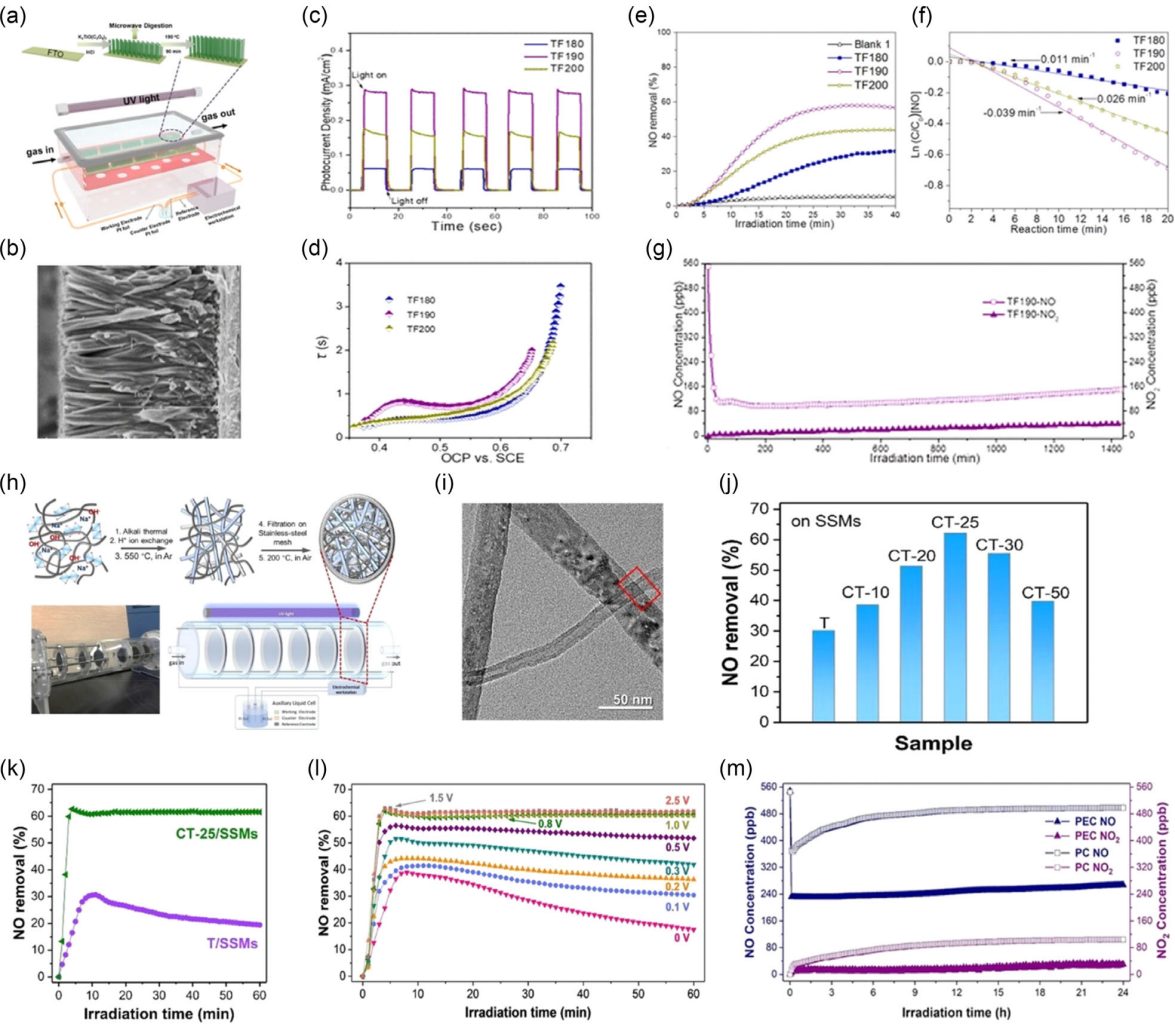
(a) Schematic illustrations of the fabrication route for TiO_2_‐NRs/FTO photoanodes and the gas‐phase PEC oxidation reactor, (b) FE‐SEM images of TF190, (c) photocurrent responses of TiO_2_‐NR/FTO films under on–off light (3.5 W LED, 365 nm, 0.3 V) irradiation, (d) open‐circuit voltage decay and electron lifetimes of TF180, TF190, and TF200, (e) NO removal efficiency via the PC oxidation process, (f) dependance of ln(*C*/*C*_0_) on irradiation time, (g) durability test of TF190 for the PEC oxidation of NO. Reproduced with permission [36]. Copyright 2020, American Chemical Society, (h) schematic illustration of the fabrication route for CNT‐TiO_2_ on a SSM photoelectrode including digital photo of the PEC NO reactor, and schematic illustration of the PEC NO oxidation reactor and its working mechanism, (i) HR‐TEM images of CT‐25, (j) NO removal rates of various TiO_2_–CNT/SSMs photoanodes, (k) reaction profiles for PEC oxidation of NO under UV‐light irradiation (*λ* = 365 nm, 8 × 6 W), (l) PEC oxidation of NO gas with the CT‐25/SSM photoanode via applying different bias voltages (0 to 2.5 V) under UV‐light (*λ* = 365 nm) irradiation and (m) stability of PEC and PC NO oxidation over time. Reproduced with permission [65, 66, 67, 68]. Copyright 2019, American Chemical Society.

Daietal. introduced the first gas‐phase PEC reactor capable of highly efficiently oxidizing trace NO in indoor air while generating minimal secondary pollution [36]. The system integrates a liquid‐phase electrochemical auxiliary cell (seeing Figure 7a) with a tandem stack of ten TiO_2_ nanorod array films directly grown on fluorine‐doped tin oxide (TiO_2_‐NR/FTO) glass. Two units are connected in series, with a small bias of 0.3V versus SCE driving charge separation at the gas–liquid interface. The rutile TiO_2_ nanorods are about 3.83 μm long and approximately 200 nm in diameter (seeing Figure 7b). Among control samples grown at 180 or 200°C, the sample TF 190 exhibits the highest photocurrent density (0.28mAcm^−2^ at 365nm LED and 0.3V), the greatest donor density (10.3 × 10^30^ cm^−3^), and the longest electron lifetime (0.28s) (seeing Figures 7c‐d). This sample shows the highest photocatalytic activity for NO removal, consistent with the PEC test results (Figure 7e). After 30 min of illumination, the NO removal rate reaches 58%. The initial rates of photocatalytic NO oxidation follow the Langmuir‐Hinshelwood (L‐H) model, with a linear ln(C/C_0_) versus time plot (Figure 7f) indicating mass‐transfer‐controlled first‐order kinetics‐likely due to very low indoor NO concentrations. When 500ppb of NO flows at room temperature, TF 190 removes 58% of NO in 30 min; applying a 0.3V bias boosts removal to 82%, and the rate constant doubles to 0.085 min^−1^. Increasing the bias to 0 5V reduces activity because too wide a space‐charge layer promotes self‐quenching of ·OH radicals. The reactor functions well with both dry and 70% humidity streams; humidity adds an approximately 10% increase in conversion by supplying water for radical formation. A durability test (seeing Figure 7g) confirms stable performance, with NO_2_ byproduct levels remaining near detection limits (<a few ppb) under humid conditions. These results demonstrate that this FTO‐based gas‐phase PEC system is a durable and practical reactor for continuous NO_*X*_ treatment.

Xiaoetal. developed a gas‐phase PEC system that can selectively convert trace nitric oxide (NO, 550ppb) into harmless nitrate at room temperature. This addresses a key challenge in PEC processes—the low conductivity of air. Their approach involves placing a specially designed 3D photoanode directly in the gas stream, connected to a separate liquid‐phase cathode to complete the circuit. The photoanode is fabricated by filtering a slurry of porous anatase TiO_2_ nanoribbons and ultra‐long carbon nanotubes (CNTs) onto a stainless‐steel mesh (SSM), followed by gentle annealing (seeing Figure 7h) [68]. Six such meshes are mounted in parallel inside a quartz tube, which is flushed with humidified NO‐containing air. A Pt foil immersed in 0.5M Na_2_SO_4_ serves as the liquid cathode, with the two chambers linked by an external power supply. Transmission electron microscopy (Figure 7i) shows that a 7µm thick intertwined CNT/TiO_2_ network forms good electrical contact with the SSM while maintaining high porosity. Furthermore, the product distribution is strongly influenced by the balance between photogenerated holes and reactive oxygen species (ROS). In the TiO_2_ nanorod/FTO and CNT–TiO_2_ mesh photoanodes demonstrate that selectivity toward NO_3_^−^ is governed by efficient electron–hole separation, prolonged hole lifetimes, and minimized self‐quenching of ·OH radicals. Controlled bias (typically 0.3–0.8 V) is critical; too low suppresses activity, while too high accelerates radical recombination and reduces NO to NO_3_^−^ conversion. Mechanistic studies reveal that ·OH, ·O_2_^−^, and ^1^O_2_ act synergistically, with ·OH enabling initial NO to HONO/NO_2_ formation and ·O_2_^−^/^1^O_2_ promoting deeper oxidation to NO_3_^−^. Importantly, the catalyst structures such as ordered nanorods, single‐atom active sites (Pt, Zn, Ag), or heterojunctions provide tunable adsorption strengths and preferential ROS pathways that suppress toxic NO_2_ byproducts and enhance nitrate selectivity. Optimized cocatalysts (e.g., Pt, Ni‐based) and interface engineering also ensure that oxidation proceeds selectively at the photoanode while protons are reduced cleanly to H_2_ at the cathode.

## 5 Computational Tools for Analyzing PEC Fuel Generation

Computational analysis has become an indispensable tool in advancing catalysis, particularly for multielectron reactions such as UOR, AOR, and the oxidation of NO_*X*_. Among various techniques, density functional theory (DFT) has played a pivotal role by providing atomic‐level insights into adsorption energetics, electronic charge transfer processes, and the stability of reaction intermediates. These analyses not only identify the rate‐determining steps but also clarify the underlying reaction pathways, enabling the rational design of catalysts with enhanced activity, selectivity, and long‐term stability. Furthermore, coupling DFT with microkinetic modeling and machine‐learning‐assisted screening has recently emerged as a powerful strategy to accelerate catalyst discovery and to bridge the gap between theoretical predictions and experimental performance [69,70].

For UOR, DFT calculations have clarified that the C—N bond cleavage step is often the main kinetic bottleneck. For example, studies on NiO@Ni/n‐Si demonstrated that surface Ni^4+^=O coverage lowers the C–N cleavage barrier from 0.74 to 0.41 eV, accelerating reaction kinetics by over two orders of magnitude. Similarly, Ni_2_P/TiO_2_ nanotube arrays showed stronger urea adsorption (E_ads_ ≈–0.78 eV) compared to Ni(OH)_2_ (–0.62 eV), with spontaneous N–H dehydrogenation and reduced CO_2_ desorption barriers (0.19 vs. 0.53 eV), thereby alleviating poisoning effects. These examples illustrate how tailoring the catalyst composition and surface chemistry can significantly enhance efficiency and selectivity.

In the case of AOR, DFT studies consistently identify the first dehydrogenation step (NH_3_ → *NH_2_) as the rate‐determining barrier. On NiCuO_*X*_/Ni/Si, it can be known that Cu sites stabilize NH_3_ adsorption, while cooperative Cu–Ni pathways lower the barrier associated with N—O bond formation (0.04  vs. 0.15 eV), explaining the experimentally observed faster kinetics and suppression of competing OER. On Pt surfaces, facet‐dependent behavior has been observed: Pt(100) preferentially stabilizes *NH_2_ coverage, favoring associative *NH_2_–*NH_2_ coupling toward N_2_, while Pt(111) binds too strongly intermediates and tends toward NO_*X*_ byproducts. These mechanistic insights provide clear guidelines for improving both activity and selectivity.

For NO_*X*_ oxidation, computational analysis showed the complexity of competing pathways (NO → HONO → NO_2_^−^/NO_3_^−^) strongly governed by reactive oxygen species and surface hydroxyls. DFT‐based reaction studies indicate that catalyst selectivity can be tuned by stabilizing HONO and nitrate intermediates, while suppressing undesired side routes. While DFT provides detailed mechanistic insights, advanced computational tools are required to more rapidly explore the vast chemical space of potential photoanodes. Here, machine learning (ML), artificial intelligence (AI), and big data analytics are emerging as powerful approaches. ML algorithms trained on DFT datasets can rapidly screen descriptors such as adsorption energy, *d*‐band center, and charge density to predict catalyst performance across large material libraries. For UOR, ML‐assisted models can identify precisely oxide or phosphide surfaces with optimized binding for urea‐derived intermediates, while in AOR, AI‐driven reaction network analysis maps dominant intermediates (*NH_2_, NO_2_^−^, NO_3_^−^) to improve selectivity. In NO_*X*_ oxidation, coupling ML models with in situ spectroscopy enables adaptive predictions that refine mechanistic insights under realistic conditions [71, 72].

Future research will focus on creating hybrid DFT–ML frameworks that optimize both accuracy and efficiency, building databases of adsorption energies and reaction barriers for nitrogen intermediates, and using generative AI to suggest new cocatalyst structures based on stability and cost considerations. Combining DFT with data‐driven approaches will speed up catalyst discovery and support the rational design of effective, selective, and long‐lasting PEC photoanodes for nitrogen oxidation reactions (Tables 2 and 3).

**TABLE 2:** Comparison table of photoanodes used for PEC UOR.

**Potential** **(*V***_**RHE**_ **)**	**Photocurrent** **density (mA·cm** ^**−2**^ **)**	Stability (h)	FE (%)
1.39	10	12	—
1.34	100	18	—
1.3	1.57	—	—
1.23	3.44	1	—
1.23	1.1	1	90.2% (H_2_)
1.23	4.60	—	—
1.23	31	1.30	—
1.08	100	1	92% (H_2_)
1	30.5	—	—
1.45	7	—	—
1.23	1.2	1	92% (H_2_)
1.93	10	—	75% (N_2_)

**TABLE 3:** Comparison table of photoanodes used for PEC AOR and NO oxidation.

**Potential** **(*V***_**RHE**_ **)**	**Photocurrent** **density (mA·cm** ^**−2**^ **)**	Stability (h)	FE (%)
1.23	1.3	3	54.4
1.23	2.81	2	80% (H_2_)
1	1.78	1	92% (H_2_) 90% (N_2_)
1	1.2	1	96% (H_2_) 95% (N_2_)
1.23	6	1	81.5% (N_2_)
1.38	12	1	99% (NO_*x*_)
PEC NO oxidation			NO elimination [%]
0.3	—	10	80%
0.3	0.3	1	85%
0.3	0.5	24	50%

## 6 Summary and Outlook

Utilizing solar energy to generate clean hydrogen holds significant promise for a sustainable, low‐carbon future. Yet, traditional PEC water splitting is hindered by the slow, energy‐intensive OER, which yields limited economic value from its oxygen byproduct. This review explores an alternative: replacing OER with the selective oxidation of urea, ammonia, and nitrogen oxides (NO_*X*_). These reactions lower energy requirements, speed up processes, and produce valuable coproducts like nitrogen gas, carbon dioxide, or nitrates—transforming waste and pollutants into resources. Recent studies in photoanode technologies are noteworthy. Engineers have developed nanostructured TiO_2_ with Ni_2_P co‐catalysts, MIS‐type Si electrodes with Ni–Fe and Ni–Mo–O overlays, and core–shell NiO@Ni structures. These innovations enhance light absorption, improve charge separation, and reduce recombination, resulting in higher photocurrents at lower voltages. This makes PEC hydrogen production more efficient and affordable, while also addressing environmental concerns—such as treating urea‐rich wastewater or capturing air pollutants like NO. Gas‐phase PEC reactors have demonstrated the ability to convert low‐level NO pollution into useful nitrates under mild conditions, with designs that control humidity and avoid secondary pollutants like NO_2_. Despite these advances, challenges remain. Catalyst stability over continuous operation is vital, as many systems degrade over time due to surface poisoning or corrosion. Ensuring selectivity is also critical—preventing partial oxidation products like nitrates from accumulating during urea oxidation, or achieving complete conversion of NO without forming toxic intermediates. Scaling up for real‐world use demands durable, low‐cost materials capable of functioning in varied environments, from wastewater to polluted urban air. Despite rapid advances in PEC nitrogen valorization, critical challenges remain in stability, selectivity, and scalability. Addressing these issues will require an integration of mechanistic insight, material design, and device engineering. Stability can be enhanced through stability‐by‐design strategies such as ultrathin oxide overlayers and hydrophobic coatings that mitigate biofouling and salt deposition while preserving charge transport. Long‐term durability should be benchmarked under realistic conditions (e.g., wastewater feeds) with quantitative metrics such as the S‐number (moles of nitrogen converted per mole of catalyst lost). Patterned cocatalyst architectures on MIS–Si electrodes also offer opportunities to localize rate‐determining steps, suppress recombination, and reduce crossover.

Selectivity may be improved through dynamic operation strategies, including programed voltages or pulsed light, which can modulate reactive oxygen species (•OH, ^1^O_2_, •O_2_^−^) and active site coverage in real time. These approaches provide a route to steer reactions toward benign products such as N_2_ while suppressing undesired byproducts (HONO, NO_2_, NO_3_^−^). Operando spectroscopies (XAS, Raman, ESR) combined with ^15^N isotope labeling will be crucial to validate these pathways and uncover mechanistic origins of selectivity. Scalability requires progress beyond batch reactors toward advanced flow‐based PEC devices such as gas‐diffusion electrodes and bipolar‐membrane systems. These designs can decouple pH environments, lower ohmic resistance, and allow modular integration with photovoltaic sources. Techno‐economic and life‐cycle analyses must accompany such innovations to ensure cost‐effectiveness and environmental sustainability. Complementary strategies, including buffer and mediator design (e.g., phosphate or borate buffers, TEMPO mediators), chloride‐tolerant operation with selective membranes or scavengers, and humidity‐programed interfaces for gas‐phase NO_*X*_ systems, may further expand operational windows for stability and selectivity. Overall, PEC nitrogen valorization will advance most effectively by combining stability engineering, dynamic selectivity control, and scalable device design, thereby establishing a roadmap toward efficient, selective, and sustainable solar‐to‐chemical energy conversion.

In conclusion, adopting a new approach to the anode reaction expands the scope of PEC technologies beyond simple water splitting, enabling the conversion of waste into resources, the purification of air and water, and the production of sustainable solar fuels. Realizing this potential requires continued innovation in materials science, reactor design, and system integration, but the benefits for clean energy and environmental health are substantial and worth pursuing.

## Author Contributions

**Maheswari Arunachalam**: formal analysis (lead), investigation (lead). **Jyoti Ganapati Badiger**: investigation (equal), methodology (equal). **Suzan Abdelfattah Sayed**: validation (equal). **Soon hyung Kang**: supervision (lead).

## Funding

This work was supported by the National Research Foundation of Korea (2018R1A6A1A03024334, RS‐2025‐00523371), Korea Basic Science Institute (RS‐2025‐024130294), Ministry of Education.

## Conflicts of Interest

The authors declare no conflicts of interest.
